# Total Synthesis of Resveratrone and *iso*‐Resveratrone

**DOI:** 10.1002/open.202200098

**Published:** 2022-06-30

**Authors:** Stefan Fritsch, Nazli Aldemir, Jan Balszuweit, Kevin Bojaryn, Jens Voskuhl, Christoph Hirschhäuser

**Affiliations:** ^1^ Organic Chemistry and Center for Nanointegration Duisburg-Essen (CeNIDE) University of Duisburg-Essen Universitätsstr. 5–7 45117 Essen Germany

**Keywords:** epoxide, fluorescence label, olefination, resveratrone, total synthesis

## Abstract

The first total synthesis of resveratrone and *iso*‐resveratrone based on an epoxide olefination approach is described. The pivotal reaction proceeds by insertion of the lithiated epoxide into a boronic ester and subsequent *syn*‐elimination. Resveratrone has been described to have remarkable photophysical properties, including two‐photon absorption. Therefore, an azide derivative has been prepared to allow for use as a biological label.

## Graphical abstract

**The first total synthesis for resveratrone** and its regioisomer with comparable photophysical properties is reported. The key step involves a novel epoxide olefination and the route is applied to the synthesis of a click‐ready fluorescence label. The pivotal olefination step relies on insertion of the lithiated epoxide into the boronic ester, after which a thermic *syn*‐elimination exclusively furnishes the *trans*‐alkene. The twofold excess of boronic ester, usually required for this olefination, could be reduced to 1.3 equivalents for aryl boronates without loss of yield.

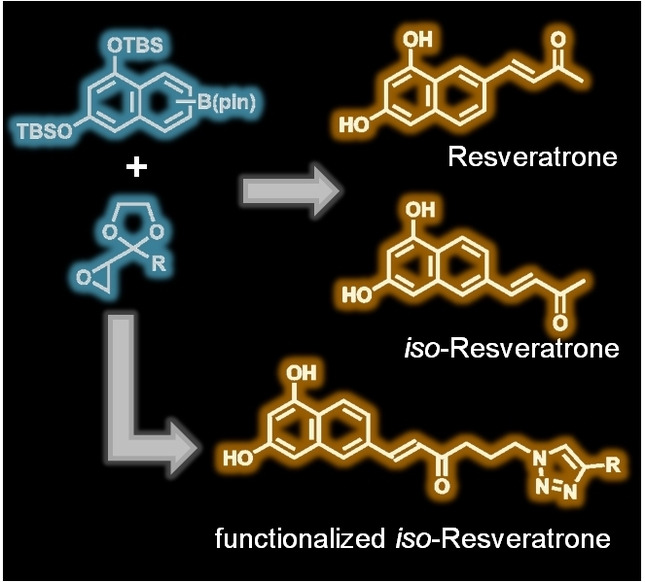

## Introduction

**Scheme 1:**
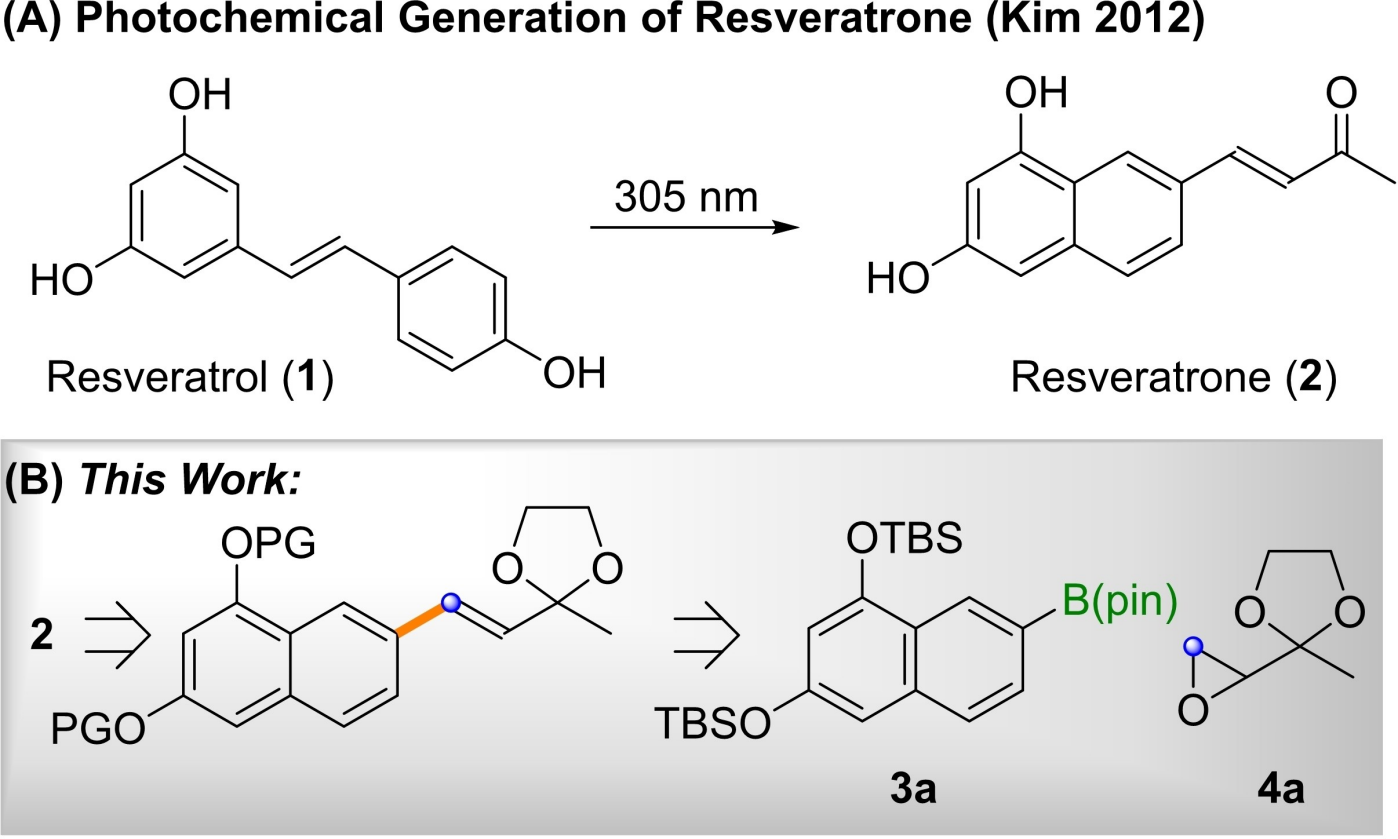
Resveratrone (**2**) from resveratrol (**1**) and key disconnection (PG=protecting group, TBS=*tert*‐butyldimethylsilyl).

Resveratrol (**1**) is a naturally occurring phenol produced by a variety of plants in response to external pathogens.^[1]^ It has gained some notoriety as a potentially “healthy” ingredient in red wine,^[2]^ although resveratrol's lifespan‐enhancing effects are strongly debated.^[3]^ In 2012, Kim and co‐workers have reported that resveratrol (**1**) reacts to resveratrone (**2**) under UV irradiation as shown in Scheme 1A.^[4]^ Resveratrone (**2**) is a highly fluorescent compound that can undergo two‐photon absorption, making it interesting for bio‐labelling applications.[^4a^, ^5^] More recently, the photo‐switchability between **1** and **2** has been utilized by Voskuhl, Giese and co‐workers for the preparation of light‐responsive liquid crystals.^[6]^ In his 2012 publication, Kim stated: “*It will be interesting to see if this relatively simple molecule can be synthesized without resorting to a photochemical reaction*”. ^[4a]^ Beyond this challenge, a total synthesis of resveratrone (**2**) appeared desirable as it provides opportunities for structural modification and might avoid the extensive late stage purification by advanced chromatography techniques, which is necessary when **2** is prepared directly from **1**.

**Scheme 2:**
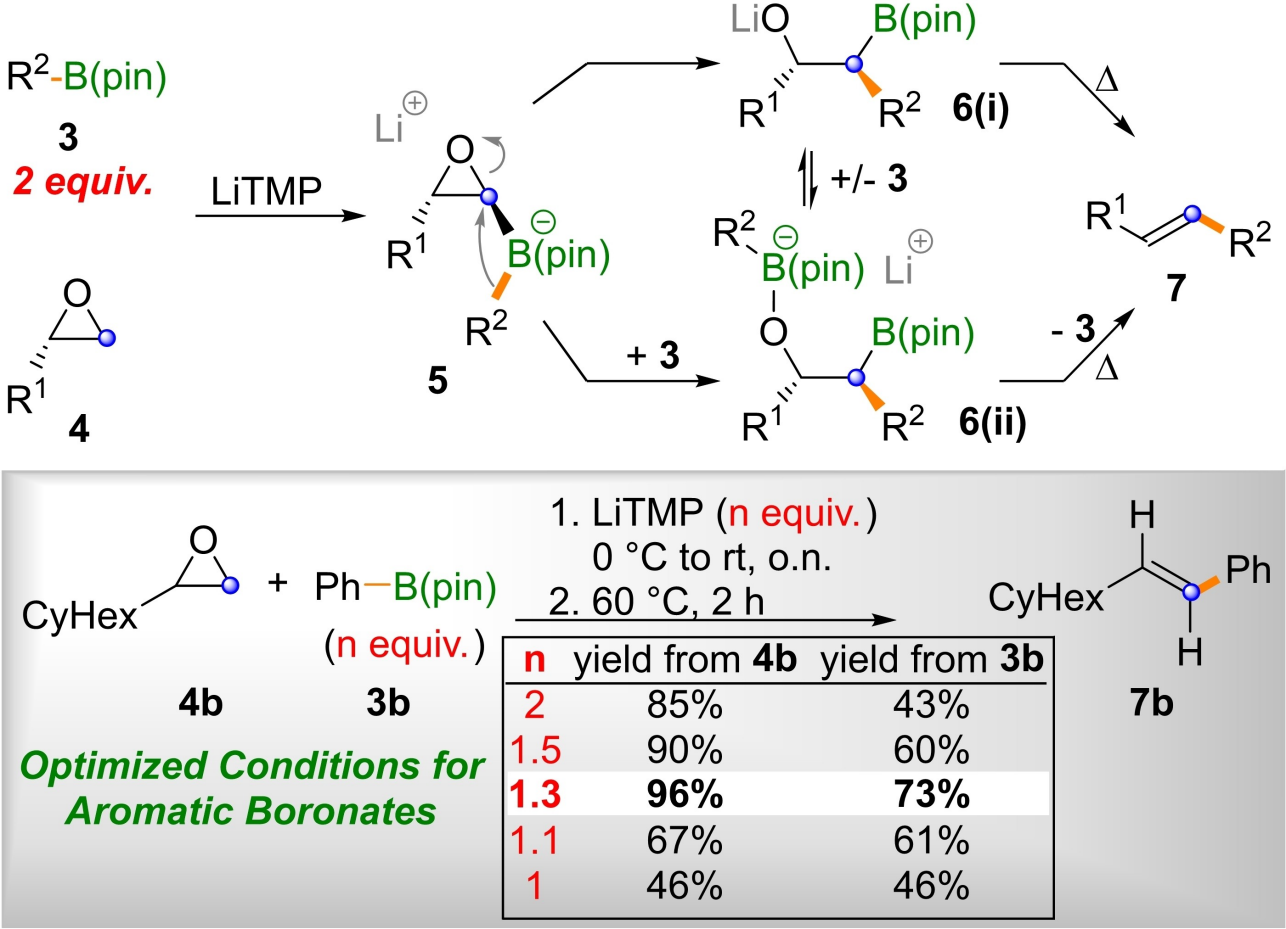
Epoxide olefination and optimization of reaction conditions for aromatic boronates (LiTMP=lithium tetramethylpiperidide, pin=pinacol).

For reasons of stability, we assumed that a protected resveratrone derivative, in which the acidic character is suppressed, would be a desirable precursor (Scheme 1B). For the pivotal formation of the aryl‐vinyl bond, we envisioned the use of an epoxide olefination, which would require boronate **3 a** and the literature‐known epoxide **4 a** ^[10]^ as suitable precursors. The epoxide olefination method employed was reported by us in 2019,^[9]^ utilizing epoxides such as **4**, or even their more highly substituted congeners. Epoxides of type **4** can be lithiated in the presence of boronic esters, ^[7]^ thus forming *ate*‐complexes (**5**), which can undergo 1,2‐metallate rearrangements to β‐alkoxy boronates (**6**; Scheme 2).^[9]^ Stereospecific *syn*‐elimination delivers alkenes of type **7** as well as their tri‐ and tetrasubstituted congeners upon iterative application. The synthesis of resveratrone sets up an interesting challenge for this method, as it usually requires the use of two equivalents of the boronic ester **3**. As, in this case, the boronic ester would be the valuable 1,3,7‐substituted dinaphthol **3 a**, this was not attractive. We thus first set out to optimize the epoxide olefination for aromatic boronates of type **3** on the simple test system **7 b**.

## Results and Discussion

**Scheme 3:**
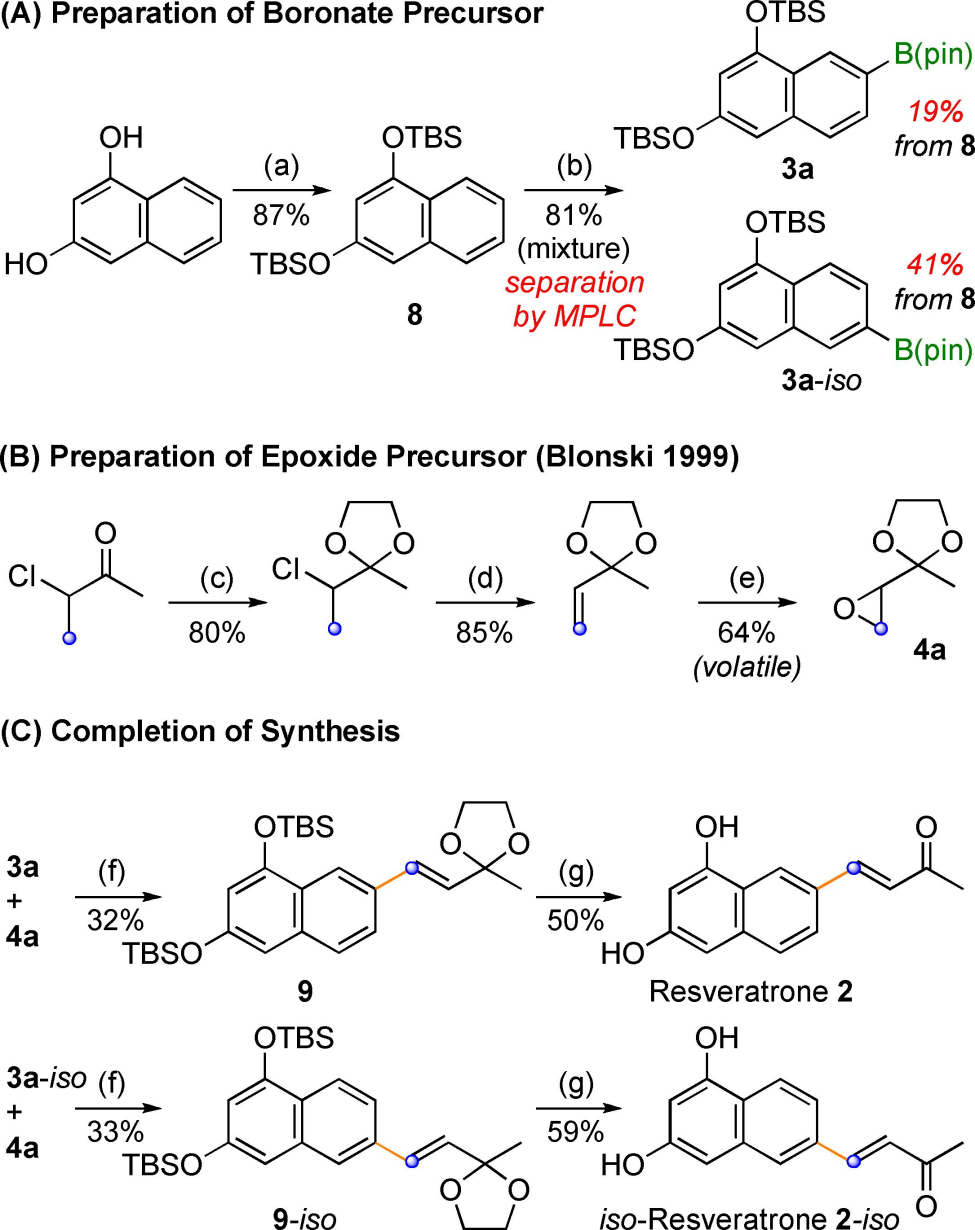
(a) TBSCl (3.0 equiv.), imidazole (3.5 equiv.), DCM, rt, 16 h; (b) B_2_Pin_2_ (2.0 equiv.), [Ir(COD)(OMe)]_2_ (0.04 equiv.), dtbpy (0.06 equiv.), *n*‐hexane, reflux,16 h; (c) *p*TsOH (0.02 equiv.), (HOCH_2_)_2_, Ph−H, 80 °C, 16 h; (d) KOH, (HOCH_2_)_2_, 130 °C, 3 h; (e) Oxone (5 equiv.), NaHCO_3_ (10 equiv.) acetone, 3 d; (f) **9** (1.3 equiv.), LiTMP (2.0 equiv.), THF 0 °C to rt, overnight, 60 °C, 2 h; (g) 1. TBAF, THF, 0 °C, 3 h, 2. HCl (2 m in H_2_O), THF, overnight, rt (COD=cyclooctadiene, dtbpy=4,4′‐di‐*tert*‐butyl‐2,2′‐dipyridyl).

Our earlier attempts to reduce the amount of alkylboronic ester necessary for epoxide insertion below two equivalents were unsuccessful.^[9]^ This was attributed to the formation of ate complexes such as **6(ii)**, which could arise either directly from **5** – excess boronate facilitates epoxide opening – or by simple complexation of the alkoxide **6(i)**. For R^2^=alkyl, this leads to the consumption of two equivalents of boronic ester per lithiated epoxide. However, for aromatic boronates, the subsequent elimination occurs comparatively swiftly, so that excess boronate **3** might be re‐liberated before the lithiated epoxide decomposes. Thus, by slowly raising the temperature overnight, the required excess of phenyl boronic ester **3 b** was reduced substantially and the best results for the synthesis of alkene **7 b** from cyclohexyl oxirane **4 b** were obtained when 1.3 equivalents of **3 b** were employed. Based on these encouraging results, we set out to synthesize resveratrone **2** as shown in Scheme 3.

For this, the required 1,3,7‐dinaphthol derivative **3 a** was prepared as shown in Scheme 3A. TBS protection of 1,3‐dinaphthol^[11]^ yielded **8** and subsequent Hartwig‐Miyaura borylation delivered **3 a**.^[12]^ Both reactions proceeded in excellent yield, but, as expected, borylation of **8** furnished a 1 : 1 mixture of **3 a** and its isomer **3 a**‐*iso*. Separation of the regioisomers was readily achieved by MPLC (RP^18^, MeOH/H_2_O 9 : 1) and their structures were assigned by NOESY NMR experiments (see Supporting Information). Interestingly, **3 a** started to decompose under MPLC conditions,^[13]^ while its regioisomer **3 a**‐*iso* remained intact. While this reduced the yield of **3 a** to 19 %, isolating **3 a**‐*iso* with a purity of more than >90 % became reasonably easy this way.

In order to complete the synthesis of resveratrone, the required epoxide **4 a** was prepared as described by Blonski and co‐workers (Scheme 3B).^[10]^ As shown in Scheme 3C, epoxide olefination to **9** and **9**‐*iso* as well as subsequent deprotection to resveratrone (**2**) and *iso*‐resveratrone (**2**‐i*so*) proceeded in moderate yields.

Given the instability of **3 a** under MPLC conditions and the resulting ease with which **3 a**‐*iso* can be obtained, we decided to briefly investigate the photophysical properties of *iso*‐resveratrone (**2**‐*iso*) in comparison to resveratrone (**2**).

**Figure 1:**
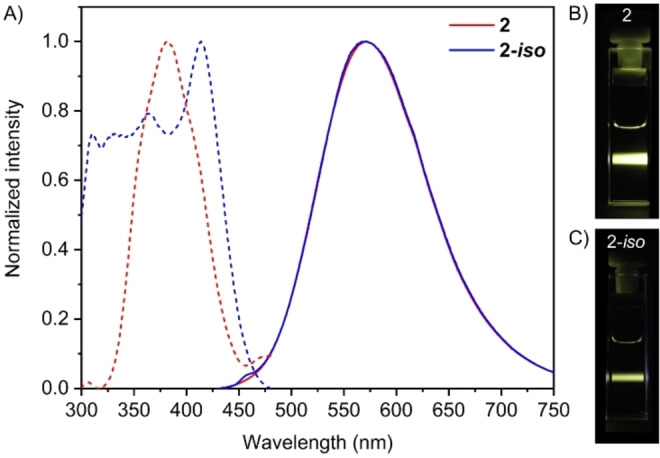
A) Excitation (dashed) and emission (solid) spectra of **2** and **2**‐*iso* in methanol, c=100 μm. B) 100 μm **2** in MeOH under UV light excitation (*λ*_ex_=405 nm), C) 100 μm **2‐*iso*** in MeOH under UV light excitation (*λ*_ex_=405 nm).

It was found that both compounds **2** and **2**‐*iso* showed quite similar emission profiles, which were in good agreement with those reported in literature. Both compounds revealed the same emission maximum at 571 nm irrespective of their slight deviations in excitation spectra (Figure 1).

**Scheme 4:**
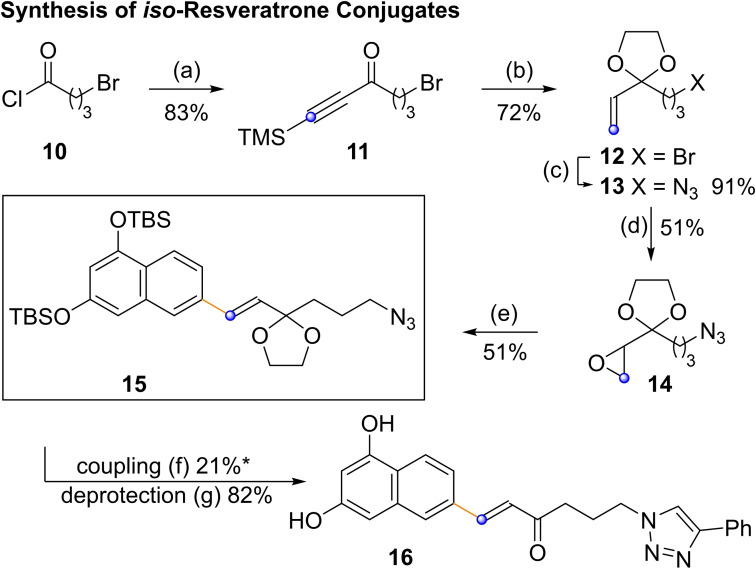
(a) AlCl_3_ (1.30 equiv.), (TMSC)_2_, DCM, 0 °C to rt; (b) 1. (HOCH_2_)_2_, pTsOH (0.07 equiv.), 16 h, 85 °C, 2. TBAF, THF, 1 h 0 °C to rt, 3. Lindlar cat. (0.05 equiv.), H_2_ (1 atm.), pyridine, 16 h, rt. (c) NaN_3_ (3.00 equiv.), DMF, 16 h, rt; (d) oxone (5.00 equiv.) NaHCO_3_ (10 equiv.), acetone, 0 °C to rt, 4 d; (e) LiTMP (2.00 equiv.), **3 a**‐*iso* (1.50 equiv.) 0 °C to rt 24 h, 60 °C 2 h; (f) CuSO_4_ (0.10 equiv.), sodium ascorbate (0.20 equiv.), THF, H_2_O, 2 d, rt; (g) HCl^aq^ (1 m), THF, 16 h, rt. (*) Yield of click product without deprotected side products.

Having established a reliable route to this interesting fluorophore and its photochemically related regioisomer, we applied it to the synthesis of the azide‐functionalized derivative **15**, which we identified as a potentially interesting compound for molecular labelling (Scheme 4). Starting from acid chloride **10**, an AlCl_3_‐mediated reaction with bis‐TMS‐acetylene yielded **11** in 83 % yield. Acetal cleavage, desilylation and Lindlar reduction required only minimal workup. Bromide **12** was isolated in 72 % overall yield, before reaction with NaN_3_ delivered **13** in 91 % yield. Epoxidation with oxone delivered the epoxide required for olefination in 51 % yield.

Given the ease with which **3 a**‐*iso* can be prepared combined with the related photochemical properties of *iso*‐resveratrone (**2**‐*iso*) and resveratrone (**2**), olefination was conducted with 1.5 equivalents of **3 a**‐*iso*. The suitably protected, click‐ready fluorophore **15** was obtained in 51 % yield. Coupling and deprotection of **15** was tested with phenylacetylene. However, under aqueous click‐coupling conditions,^[14]^ partial cleavage of the TBS groups took place, leading to only 21 % of the click‐product being isolated, deprotection of which yielded **16**.

## Conclusions

In summary, we have reported the first total synthesis of resveratrone using the epoxide olefination as a key transformation. The amount of necessary boronate for the epoxide olefination can be reduced to 1.3 equiv. for aromatic boronates (**3**), which is a vital improvement for sequences that require the coupling of valuable boronic esters such as **3 a**. A reoccurring challenge in the preparation of such substituted naphthalene derivatives lies in the lack of selectivity upon functionalization of 1,3‐dinaphthol derivatives.^[15]^ This was also seen in the preparation of **3 a**. However, separation from the regioisomer **3 a**‐*iso* was achieved by MPLC. Partial decomposition of **3 a** made **3 a**‐*iso*, and thus *iso*‐resveratrone (**2**‐*iso*), more readily accessible. First photochemical experiments point towards a comparable behavior of **2**‐*iso*, so we applied our route to the synthesis of the click‐ready *iso*‐resveratrone derivative **15**, which will hopefully serve the community as a useful labelling tool.

## Conflict of interest

The authors declare no conflict of interest.

1

## Supporting information

As a service to our authors and readers, this journal provides supporting information supplied by the authors. Such materials are peer reviewed and may be re‐organized for online delivery, but are not copy‐edited or typeset. Technical support issues arising from supporting information (other than missing files) should be addressed to the authors.

Supporting Information

## Data Availability

The data that support the findings of this study are available in the supplementary material of this article.
