# Supplementary material for: Total Synthesis of Resveratrone and iso‐Resveratrone
Source: ChemistryOpen. 2022 Jun 30;11(7):e202200098. doi: 10.1002/open.202200098 (PMC9278093; doi:10.1002/open.202200098)
Supplement: Supplementary file 1 — Supporting Information [file OPEN-11-e202200098-s001.pdf]

ChemistryOpen

Supporting Information

Total Synthesis of Resveratrone and *iso*-Resveratrone

Stefan Fritsch, Nazli Aldemir, Jan Balszuweit, Kevin Bojaryn, Jens Voskuhl,* and Christoph Hirschhäuser*

Authors and contributions

S. Fritsch: Optimization of olefination conditions for aromatic substrates, application to the synthesis of Resveratrone and *iso*-Resveratrone, first synthesis of clickable iso-Resveratrone-Conjugate.

N. Aldemir: Synthesis of *iso*-Resveratrone-Conjugate, click reaction with Phenylazide.

K. Bojaryn: Initial development of the olefination procedure and manuscript preparation.

J. Balszuweit: Photophysical measurements.

Jun.-Prof. Dr. J. Voskuhl*: Project conception and Manuscript preparation

Dr. C. Hirschhäuser*: Project conception, supervision and manuscript preparation

1 Content

2 General Experimental Conditions 2

3 Procedures 3

3.1 Preparation of Boronic Esters 3a and 3a-*iso* 3

3.1.1 (Naphthalene-1,3-diylbis(oxy))bis(*tert*-butyldimethylsilane) **8** 3

3.1.2 Naphthalene boronate **3a**/ **3a**-*iso* 4

3.2 Synthesis of Epoxide 4a 6

3.2.1 2-(1-Chloroethyl)-2-methyl-1,3-dioxolane (**S1**) 6

3.2.2 2-Methyl-2-vinyl-1,3-dioxolane (**S2**) 6

3.2.3 2-Methyl-2-(oxiran-2-yl)-1,3-dioxolane (**4a**) 7

3.3 Synthesis of Resveratrone and iso-Resveratrone 8

3.3.1 Protected Resveratrone Derivative **9** 8

3.3.2 Resveratrone (**2**) 9

3.3.3 Protected *iso*-Resveratrone Derivative **9**-*iso* 10

3.3.4 Partial deprotection of **9**-*iso* to **S3**-*iso* 10

3.3.5 Acetale Cleavage to iso-Resveratrone **2**-*iso* 11

3.4 Synthesis of a clickable iso-Resveratrone Conjugate 12

3.4.1 7-Bromo-1-(trimethylsilyl)hept-1-yn-3-one (**11**) 12

3.4.2 2-(4-Bromobutyl)-2-vinyl-1,3-dioxolane (**12**) 13

3.4.3 2-(4-Azidobutyl)-2-vinyl-1,3-dioxolane (**13**) 14

3.4.4 2-(4-Azidobutyl)-2-(oxiran-2-yl)-1,3-dioxolane (**14**) 14

3.4.5 Clickable *iso*-Resveratrone (**15**) 15

3.4.6 Click reaction with phenylacetylene and deprotection to **16** 16

4 NMR-Spectra 17

2 General Experimental Conditions

All reactions using dry solvents were carried out under argon in glassware dried with a heat gun under vacuum. Solvents for chromatography, unless purchased as *pro analysi* (p.a.) grade, were distilled over a rotary evaporator before use. THF was always freshly distilled from sodium/benzophenone, as was the case of Et_2O, when employed for reactions. *N,N,N,N*-tetramethyl piperidine was distilled from CaH_2 and stored in a Schlenk tube under argon. *n*-BuLi was purchased as a solution and stored under argon at rt. Aged BuLi solutions were titrated against *N*-benzylbenzamide. All other reagents were used as supplied from commercial sources and stored appropriately. ^{1}H- and ^{13}C- NMR spectra were recorded in deuterated solvents on Bruker DMX 300, AV NEO 400 and Bruker DRX 500 spectrometers. IR spectra were measured on a Jasco FT/IR-430 with ATR attachment spectrometer. Low and High resolution ESI mass spectra were recorded with a Bruker amaZon SL and a Bruker maXis 4G spectrometer, respectively. Emission and excitation spectra were measured on a Shimadzu RF 6000 spectrophotometer in low volume quartz cuvettes. Methanol was used in spectroscopic grade. The photographs of the compounds UV-light (λ_{ex} = 405 nm, laserpointer) were obtained using a CANON *EOS 1100D* camera.

3 Procedures

3.1 Preparation of Boronic Esters 3a and 3a-*iso*

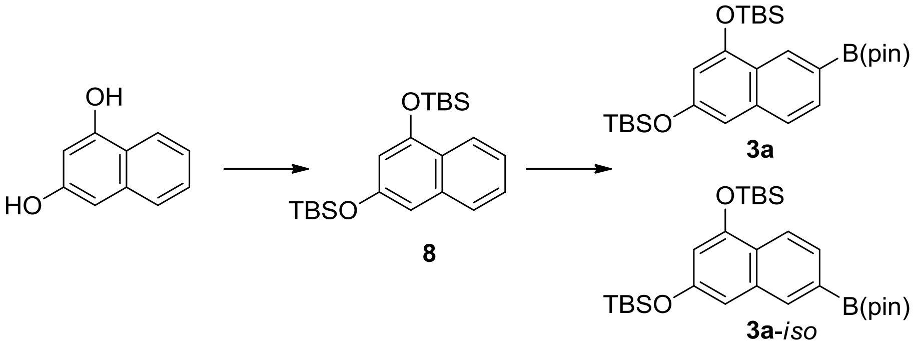

3.1.1 (Naphthalene-1,3-diylbis(oxy))bis(*tert*-butyldimethylsilane) 8

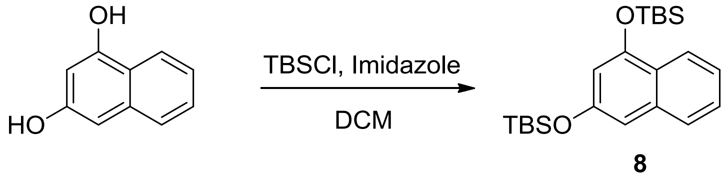

Under an atmosphere of argon naphthalene-1,3-diol (1 g, 6.24 mmol, 1.00 eq.) was dissolved in DCM (60 mL), cooled to 0 °C and imidazole (1.49 g, 21.8 mmol, 3.50 eq.) and *tert*-butyldimethylsilylchloride **5b** (2.82 g, 18.73 mmol, 3.00 eq.) were added. After stirring for 10 min at 0 °C the reaction mixture stirred for 16 h at rt. Subsequently, water (70 mL) was added and the mixture was extracted with DCM (2 x 70 mL). All organic layers were collected, washed with brine (70 mL), dried with Na_2SO_4 and the solvent was evaporated under reduced pressure. The crude product was purified by silica gel column chromatography (CyHex/EtOAc, 49:1) yielding the silyl protected diol **8** (2.12 g, 5.45 mmol, 87 %) as a yellow oil.

$\mathbf{R_f}$ = 0.77 (CyHex/EtOAc, 49:1).

^{1}H-NMR (400 MHz, $CDCl_3$): δ = 7.97 – 7.92 (m, 1H), 7.50 (d, J = 8.2 Hz, 1H), 7.26 (ddd, J = 8.2, 6.8, 1.3 Hz, 1H), 7.16 (ddd, J = 8.2, 6.8, 1.2 Hz, 1H), 6.72 (d, J = 2.1 Hz, 1H), 6.37 (d, J = 2.2 Hz, 1H), 0.96 (s, 9H), 0.89 (s, 9H), 0.16 (s, 6H), 0.11 (s, 6H) ppm.

^{13}C-NMR (101 MHz, $CDCl_3$): δ = 153.7, 152.8, 135.6, 126.8, 126.7, 124.2, 123.1, 122.71, 109.3, 108.7, 26.03, 26.0, 18.6, 18.5, -4.1, -4.1 ppm.

IR-FTR: ṽ **=** 3058, 2954, 2929, 2892, 2857, 2740, 2711, 2643, 2547, 2487, 2298, 2098, 1920, 1812, 1625, 1594, 1577, 1540, 1506, 1471,1455, 1402, 1361, 1344, 1290, 1255, 1238, 1197, 1164, 1149, 1097, 1027, 998, 937, 916, 831, 777, 746, 674, 615 cm^{-1}.

MS (ESI-pos.) *m/z* = calc. for $C_{22}H_{36}O_2Si_2$ $[M+H]^+$ 389.2327, found 389.2324.

3.1.2 Naphthalene boronate 3a/ 3a-*iso*

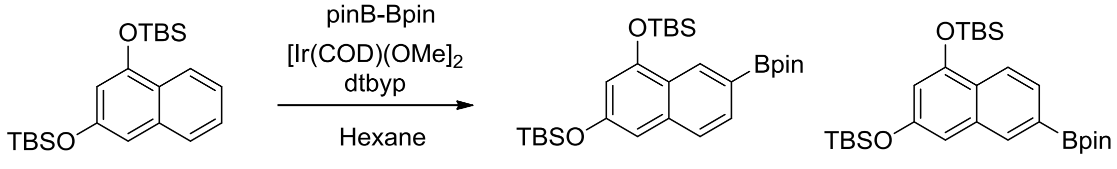

8 **3a** **3a**-*iso*

Under an atmosphere of argon TBS-diol **8** (2.00 g, 5.15 mmol, 1.00 eq.) was dissolved in dry hexane, bis(pinakolato)-diborane (2.61 g, 10.29 mmol, 2.00 eq.), bis(1,5-cyclooctadien)-dimethoxydiiridium (136 mg, 0.20 mmol, 0.04 eq.) and 4,4′-bis(2-metyhl-2-propanyl)-2,2′-bipyridine (82.5 mg, 0.31 mmol, 0.06 eq) were added to the solution. The reaction mixture was stirred for 18 h at 95 °C. Subsequently, the reaction mixture was cooled at 0°C, aqueous NH_4Cl (33 mL) and brine (30 mL) were added and extracted with EtOAc (3 x 30 mL). All organic layers were collected, dried with Na_2SO_4 and the solvent was evaporated under reduced pressure. The crude product was purified by silica gel column chromatography (CyHex/Et_2O, 49:1) to mixture of regioisomers (2.15 g, 4.14 mmol, 81 %). The separation of the regioisomers was by MPLC on C18 reversed-phase silica gel (gradient 95 % → 100 % methanol/water over 120 min). The purification resulted regioisomer **3a** (0.5 g, 0.97 mmol, 19 %) and regioisomer **3b**-*iso* (1.10 g, 2.13 mmol, 41 %) as a colorless liquid.

R_f: 0.46 (CyHex/Et_2O, 49:1).

Boronic acid ester 3a:

^{1}H-NMR (400 MHz, $CDCl_3$): δ = 8.63 (s, 1H), 7.74 (d, *J* = 9.4 Hz, 1H.), 7.60 (d, *J* = 8.2 Hz, 1H), 6.82 (d, *J* = 2.1 Hz, 1H), 6.48 (d, *J* = 2.2 Hz, 1H), 1.35 (s, 12H), 1.11 (s, 9H), 1.01 (s, 9H), 0.27 (s, 6H), 0.24 (s, 6H) ppm.

^{13}C-NMR (101 MHz, $CDCl_3$): δ = 154.9, 153.6, 137.2, 131.4, 131.2, 125.7, 123.7, 109.5, 108.7, 83.7, 26.02, 25.9, 25.0, 18.6, 18.4, -4.1, -4.2 ppm.

MS (ESI-pos.) *m/z* = calc. for $C_{28}H_{47}B_1O_4Si_2$ $[M+H]^+$ 515.3184, found 515.3188.

IR-FTR: ṽ **=** 3640, 3054, 2954, 2931, 2890, 2857, 2740, 2711, 2300, 1930, 1830, 1776, 1623, 1598, 1303, 1255, 1236, 1195, 1145, 1106, 1078, 1004, 964, 927, 877, 852, 831, 779, 730, 698, 628 cm^{-1}.

Boronic acid ester 3a-*iso***:**

^{1}H-NMR (400 MHz, $CDCl_3$): δ = 8.16 (s, 1H), 8.03 (d, J = 8.3 Hz, 1H), 7.66 (dd, J = 8.4, 1.1 Hz, 1H), 6.90 (d, J = 2.1 Hz, 1H), 6.53 (d, J = 2.2 Hz, 1H), 1.38 (s, 12H), 1.08 (s, 9H), 1.00 (s, 9H), 0.27 (s, 6H), 0.23 (s, 6H) ppm.

^{13}C-NMR (101 MHz, $CDCl_3$): δ = 153.5, 152.7, 135.0, 134.8, 127.8, 125.7, 121.8 110.7, 109.3, 84.0, 26.0, 26.0, 25.0, 18.6, 18.5, -4.1, -4.2 ppm.

MS (ESI-pos.) *m/z* = calc. for $C_{28}H_{47}B_1O_4Si_2$ $[M+H]^+$ 515.3184, found 515.3187.

IR-FTR: ṽ = 3342, 3066, 2954, 2931, 2859, 2040, 1930, 1685, 1627, 1596, 1569, 1504, 1461, 1436, 1421, 1351, 1311, 1274, 1253, 1234, 1187, 1166, 1145, 1101, 1076, 1010, 964, 939, 908, 873, 829, 779, 723, 703, 686, 669, 622, 607 cm^{-1}.

Key NOE Interactions

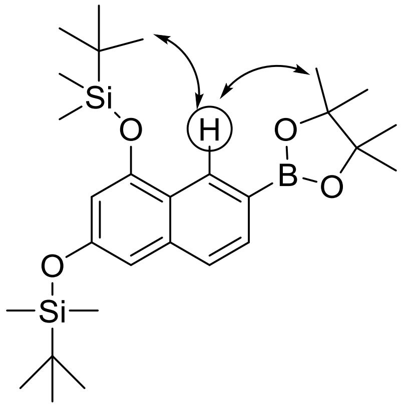

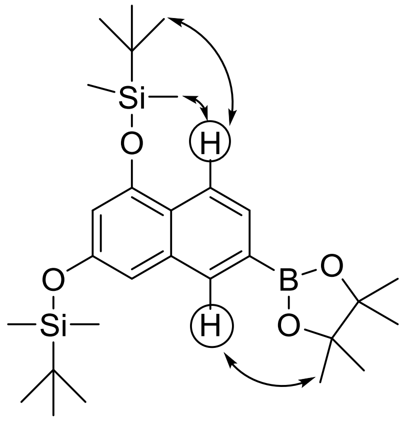

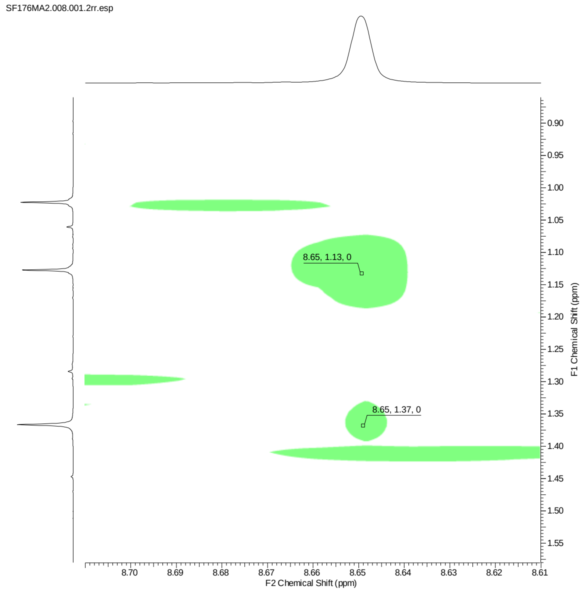

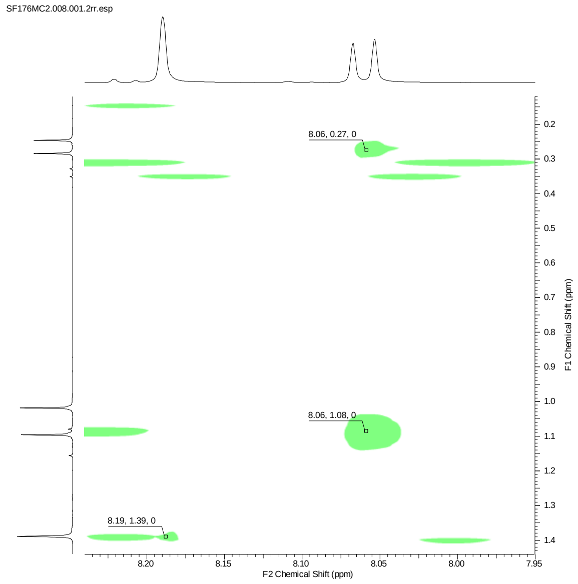

3.2 *Synthesis of Epoxide 4a*

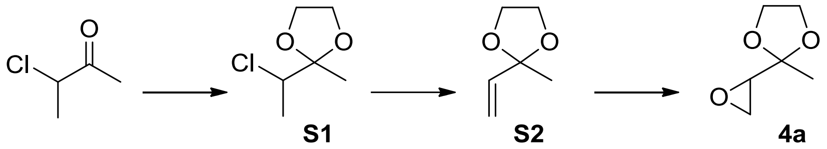

3.2.1 2-(1-Chloroethyl)-2-methyl-1,3-dioxolane (*S*1)

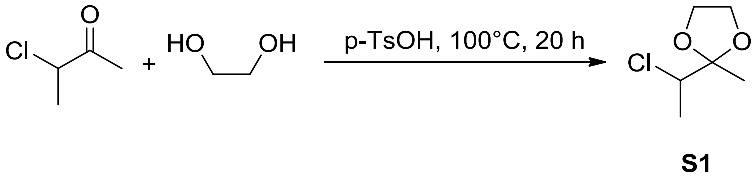

A 500 mL round bottom flask equipped with a dean stark apparatus was charged with 3-chlorobutan-2-one (23.2 mL, 0.23 mol, 1 eq.) and dissolved in benzene (330 mL) before 1,3-propandiol (16.5 mL, 0.23 mol, 1 eq.) and *p*-toluenesulfonic acid (1.00 g, 5.20 mmol, 0.02 eq.) were added. The reaction mixture was heated to reflux for 20 h. After cooling to rt the solvent was removed *in vacuo*. The product was purified by fractional distillation (13 mbar, 110 °C) from which **S1** was obtained as a colorless oil (27.8 g, 0.18 mol, 80 %).

^{1}H-NMR (300 MHz, $CDCl_3$) δ = 3.92 - 4.09 (m, 5 H), 1.52 (d, J = 6.9 Hz, 3 H), 1.44 (s, 3 H) ppm.

^{13}C-NMR (75 MHZ, $CDCl_3$): δ = 110.1, 65.6, 65.5, 60.9, 20.1, 20.0 ppm.

NMR-Data was consistent with earlier reports.[1]

3.2.2 2-Methyl-2-vinyl-1,3-dioxolane (*S*2)

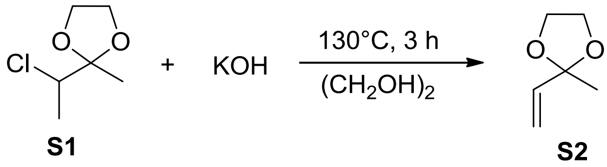

A 100 mL round bottom flask equipped with a distillation bridge was charged with KOH (22.9 g, 0.41 mol, 6 eq.) and ethylene glycol (50 mL). The reaction mixture was heated to 130 °C for 3 h, after which KOH had dissolved completely. Then **S1** (10.0 g, 66.6 mmol, 1 eq.) was added. The reaction mixture was heated to 130 °C for further 3 h. The temperature was then increased to 145 °C and the resulting alkene **S2** was isolated from the reaction mixture by distillation (bp. 85 °C). Alkene **S2** (6.44 g, 56.4 mmol, 85 %) was obtained as a colorless oil.

[1] P. Page, C. Blonski, J. Périé, *Bioorg. Med. Chem.* **1999**, 7, 7, 1403-1412.

^{1}H-NMR (300 MHz, $CDCl_3$) δ = 5.80 (dd, J = 17.2, 10.6 Hz, 1 H), 5.38 (dd, J = 17.2, 1.6 Hz, 1 H), 5.14 (dd, J = 10.6, 1.9 Hz, 1 H), 3.87 - 4.00 (m, 4 H), 1.47 (s, 3 H) ppm.

^{13}C-NMR (75 MHz, $CDCl_3$): δ = 138.4, 114.8, 107.4, 64.5, 24.6 ppm.

*NMR-Data was consistent with earlier reports.*1

3.2.3 2-Methyl-2-(oxiran-2-yl)-1,3-dioxolane (4a)

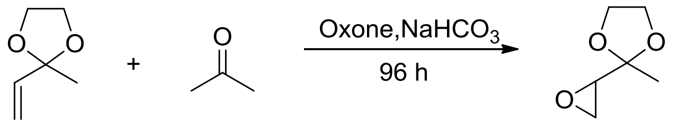

S2 **4a**

Alkene **S2** (2.00 g, 17.5 mmol, 1 eq.) was dissolved in acetone (125 mL) and cooled to 0 °C and $NaHCO_3$ (14.6 g, 17.5 mmol, 1 eq.) was added to the stirred solution. The reaction flask was equipped with a dropping funnel, over which an aqueous solution of potassium peroxysulfate (Oxone) (26.9 g, 87.0 mmol, 5 eq. in 90 mL H_2O) was added in a dropwise manner. After addition was complete, the mixture was stirred at 0 °C for 15 minutes and then for 4 days at rt. The resulting suspension was filtered, brine (50 mL) was added, and the mixture was extracted with Et_2O (2x50 mL). The combined organic layers were dried over $MgSO_4$ and the solvent was removed at the rotary evaporator, taking care to not reduce the pressure below 450 mbar as product **4a** is volatile and potentially toxic. The residue was purified by fractional distillation (bp. 30 °C at 20 mbar) yielding **4a** as a colorless oil (1.46 g, 11.2 mmol, 64 %).

^{1}H-NMR (300 MHz, $CDCl_3$) δ = 3.92 - 4.08 (m, 4 H), 3.06 (dd, J = 3.8, 2.8 Hz, 1 H), 2.69 - 2.75 (m, 2 H), 1.41 (s, 3 H) ppm.

^{13}C-NMR (75 MHz, $CDCl_3$): δ = 106.5, 66.0, 65.5, 54.9, 43.7, 21.7 ppm.

*NMR-Data was consistent with earlier reports.*1

3.3 Synthesis of Resveratrone and iso-Resveratrone

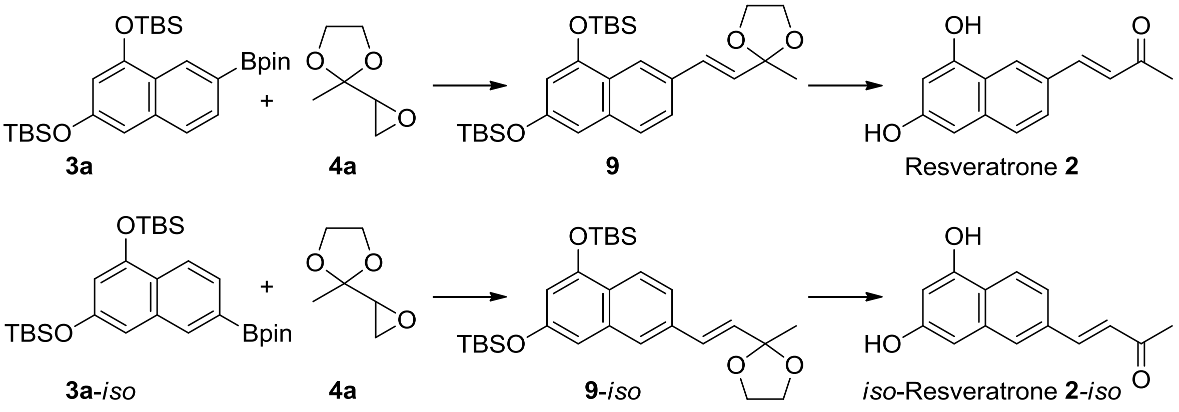

3.3.1 Protected Resveratrone Derivative 9

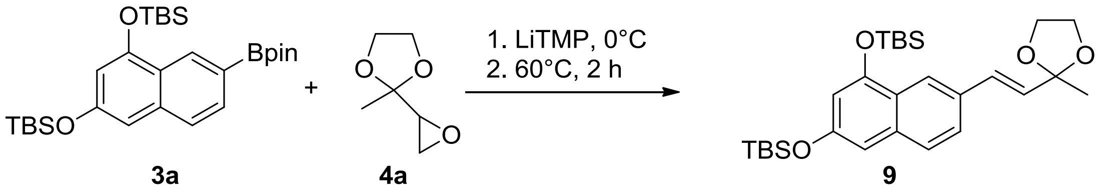

Fresh LiTMP was prepared in a dried Schlenk-tube under an atmosphere of argon by dropwise addition of *n*-BuLi (0.73 mL, 1.16 mmol, 2 eq., 1.6 M in hexanes) at 0 °C to a solution of TMP (0.22 mL, 1.28 mmol, 2.2 eq.) in dried THF (1.5 mL). The solution was allowed to warm to rt over the course of 0.5 h. In a second dried Schlenk-tube under an atmosphere of argon a solution of epoxide **4a** (75.0 mg, 0.58 mmol, 1 eq.) and boronic ester **3a** (388 mg, 0.75 mmol, 1.3 eq.) in THF (1 mL) was cooled to 0 °C. The fresh LiTMP solution was added dropwise, and the reaction mixture was stirred overnight, while the cooling bath was allowed to warm to rt. Afterwards, the solution was heated to 60 °C for 2 h. After cooling to rt, brine (15 mL) was added, followed by aqueous NaOH (1 M, 15 mL). The aqueous layer was extracted with Et_2O (3x25 mL) and the combined organic phases were dried over Na_2SO_4. The solvent was removed in vacuo and the residue was purified by column chromatography (SiO_2, CyHex/EtOAc/Et_3N 98:2:1). 92 mg (0.19 mmol, 32%) yielding **9** as a yellow oil.

R_f = 0.29 (CyHex/EtOAc, 98:2).

^{1}H-NMR (300 MHz, $CDCl_3$) δ = 7.51 - 7.62 (m, 2 H), 7.51 - 7.62 (m, 2 H), 6.80 - 6.87 (m, 2 H), 6.49 (d, *J* = 2.2 Hz, 1 H), 6.20 (d, *J* = 15.9 Hz, 1 H), 3.96 - 4.09 (m, 4 H), 1.61 (s, 3 H), 1.11 (s, 9 H), 1.02 (s, 9 H), 0.31 (s, 6 H), 0.25 (s, 6 H) ppm.

^{13}C-NMR (75 MHz, $CDCl_3$): δ = 153.9, 152.9, 135.1, 130.9, 130.3, 128.7, 127.0, 124.3, 123.9, 122.0, 109.5, 108.6, 107.8, 64.6, 25.9, 25.7, 25.3, 18.5, 18.3, -4.3 ppm.

IR-FTR: $\tilde{\nu}$ = 2954 (w), 2929 (w), 2884 (w), 2857 (w), 2483 (w), 2360 (w), 2159 (w), 2028 (w), 1976 (w), 1670 (w), 1623 (w), 1594 (m), 1571 (m), 1459 (m), 1398 (m), 1330 (m), 1292 (m),

1253 (m), 1195 (m), 1155 (m), 1085 (m), 1041 (m), 1004 (m), 937 (m), 887 (m), 827 (s), 779 (s), 730 (m), 669 (m) cm^{-1}.

MS (ESI-pos.) *m/z* = calc. for $C_{28}H_{44}O_4Si_2$ $[M+H]^+$ 501.2851, found 501.2830.

3.3.2 Resveratrone (2)

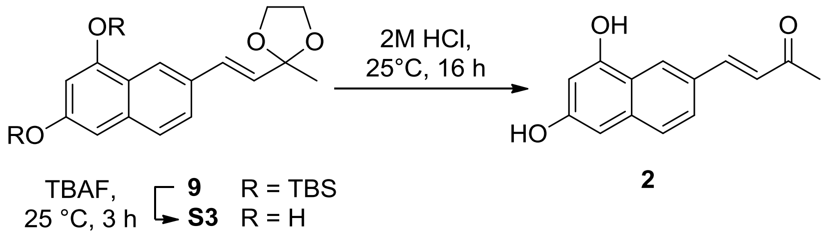

In a dried Schlenk-tube under an atmosphere of argon a solution of **9** (71.0 mg, 0.14 mmol, 1 eq.) in dry THF (0.5 mL) was cooled down to 0 °C before a solution of TBAF (0.29 mL, 0.29 mmol, 2.1 eq., 1 M in THF) was added dropwise to the solution. The reaction mixture was stirred for 3 h at rt, before dilution with Et_2O (20 mL) and extraction with aqueous, saturated NH_4Cl-solution (3x10 mL). The organic phase was dried over Na_2SO_4 und the solvent was removed *in vacuo*. The residue was purified by column chromatography (SiO_2, CyHex/EtOac/DCM/MeOH 12:4:4:1) yielding a mixture of **S3** and **2** (32 mg, 0.12 mmol, 74 %) as a yellow solid.

***Data for S3 taken from mixture*:**

$\mathbf{R_f}$ = 0.19 (CyHex/EtOAc/DCM/MeOH 6:2:2:0.5).

^{1}H-NMR (300 MHz, MeOH-d_4) δ = 7.98 (s, 1 H), 7.50 (d, J = 1.25 Hz, 2 H), 6.81 (d, J = 15.95 Hz, 1 H), 6.62 (dd, J = 2.19, 0.94 Hz, 1 H), 6.48 (d, J = 2.19 Hz, 1 H), 6.19 (d, J = 15.95 Hz, 1 H), 3.93 - 4.05 (m, 4 H), 1.55 (s, 3 H) ppm.

Complete deprotection of the mixture thus obtained (24.0 mg, 0.09 mmol) was achieved in THF (1 mL), to which HCl (0.70 mL, 2 M) was added. After stirring at rt overnight, the mixture was diluted with Et_2O (30 mL) and washed with brine (2x10 mL) and an aqueous, saturated NH_4Cl (1x10 mL). The organic layer was dried over Na_2SO_4 and the solvent was removed *in vacuo* yielding Resveratrone **2** (16 mg, 0.07 mmol, 50 % over both steps) as a yellow/orange solid.

^{1}H-NMR (300 MHz, MeOH-d_4) δ = 8.26 (s, 1 H) , 7.76 (d, J=16.3 Hz, 1 H), 7.62 (dd, J=8.8, 1.9 Hz, 1 H), 7.55 (d, J=8.8 Hz, 1 H), 6.78 (d, J=16.3 Hz, 1 H), 6.65 (d, J=1.9 Hz, 1 H), 6.52 (d, J=2.2 Hz, 1 H), 2.39 (s, 3 H) ppm.

^{13}C-NMR (75 MHz, MeOH-d_4): δ = 201.7, 159.4, 157.1, 147.2, 138.8, 129.1, 128.0, 127.1, 125.8, 125.3, 121.5, 102.3, 27.3 ppm.

NMR-Data was consistent with earlier reports.[2]

---

[2] I. Yang, E. Kim, J. Kang, H. Han, S. Sul, S. B. Park, S. K. Kim, *Chem. Commun.* **2012**, 48, 3839-3841

3.3.3 Protected *iso*-Resveratrone Derivative 9-*iso*

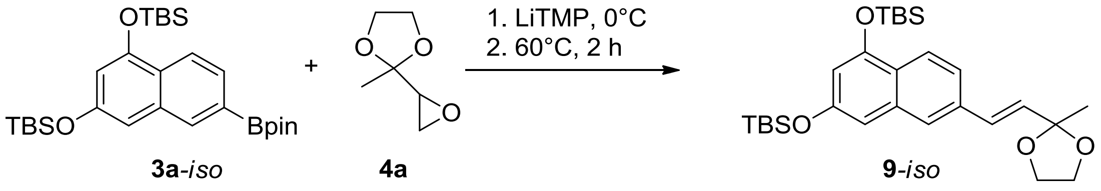

9-*iso* was prepared under the same conditions as **9** (see Section 3.3.1) using **3a**-*iso* instead of **3a**. The product was obtained as a yellow oil (96 mg, 0.19 mmol, 33%).

R_f = 0.29 (CyHex/EtOAc, 98:2).

^{1}H-NMR (300 MHz, $CDCl_3$) δ = 8.01 (d, J = 8.8 Hz, 1 H), 7.58 (s, 1 H), 7.42 (dd, J = 8.8, 1.9 Hz, 1 H), 6.79 - 6.89 (m, 2 H), 6.47 (d, J = 2.2 Hz, 1 H), 6.26 (d, J = 15.9 Hz, 1 H), 3.94 - 4.09 (m, 4 H), 1.60 (s, 3 H), 1.09 (s, 9 H), 1.02 (s, 9 H), 0.29 (s, 6 H), 0.25 (s, 6 H) ppm.

^{13}C-NMR (75 MHZ, $CDCl_3$): δ = 154.0, 152.6, 135.5, 134.4, 132.7, 130.0, 125.7, 123.0, 120.9, 109.5, 108.8, 107.7, 64.6, 25.9, 25.7, 25.3, 18.4, 18.3, -4.3 ppm.

IR-FTR: $\tilde{\nu}$ = 2954 (w), 2929 (w), 2886 (w), 2857 (w), 2520 (w), 2360 (w), 2157 (w), 2024 (w), 1976 (w), 1671 (w), 1621 (m), 1594 (m), 1571 (w), 1506 (w), 1436 (w), 1392 (m), 1340 (w), 1286 (w), 1253 (m), 1172 (m), 1153 (m), 1095 (m), 1041 (m), 1014 (m), 973 (w), 879 (s), 829 (s), 779 (s), 669 (m), 624 (m) cm^{-1}.

MS (ESI-pos.) *m/z* = calc. for $C_{28}H_{44}O_4Si_2$ $[M+H]^+$ 501.2851, found 501.2819.

3.3.4 Partial deprotection of 9-*iso* to S3-*iso*

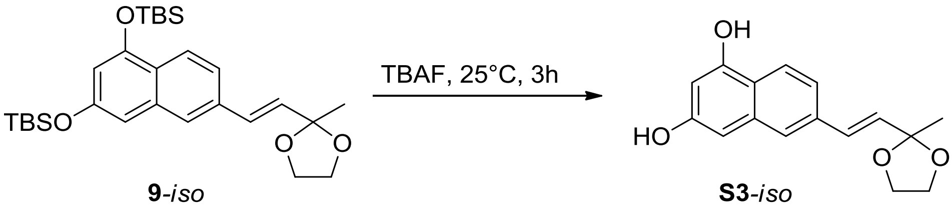

In a dried Schlenk-tube under an atmosphere of argon a solution of **9**-*iso* (83.0 mg, 0.16 mmol, 1 eq.) in dry THF (0.5 mL) was cooled down to 0 °C before a solution of TBAF (0.34 mL, 0.34 mmol, 2.1 eq., 1 M in THF) was added in a dropwise manner. The reaction mixture was stirred for 3 h at rt. The solution was then diluted with Et_2O (25 mL) and washed with aqueous, saturated NH_4Cl-solution (3x10 mL). The organic Phase was dried over Na_2SO_4 und the solvent was removed *in vacuo*. The residue was purified by column chromatography (SiO_2, CyHex/EtOac/DCM/MeOH 12:4:4:1) yielding **S3**-*iso* (32 mg, 0.12 mmol, 74 %) as a yellow solid.

R_f = 0.24 (Cyhex/EtOAc/DCM/MeOH 12:4:4:1)

^{1}H-NMR (300 MHz, MeOH-d_4) δ = 7.99 (d, J = 8.8 Hz, 1 H), 7.49 (s, 1 H), 7.34 (dd, J = 8.8, 1.6 Hz, 1 H), 6.81 (d, J = 16.3 Hz, 1 H), 6.63 (d, J = 1.9 Hz, 1 H), 6.43 - 6.49 (m, 1 H), 6.26 (d, J = 15.9 Hz, 1 H), 3.94 - 4.05 (m, 4 H), 1.55 (s, 3 H) ppm.

^{13}C-NMR (75 MHZ, MeOH-d_4): δ = 157.5, 156.0, 135.8, 131.6, 130.9, 126.5, 123.8, 120.3, 109.1, 102.1, 101.9, 65.8, 25.6 ppm.

IR-FTR: $\tilde{\nu}$ = 3291 (w), 2977 (w), 2929 (w), 2892 (w), 2541 (w), 2360 (w), 2159 (w), 2026 (w), 1976 (w), 1629 (m), 1583 (m), 1521 (w), 1446 (w), 1398 (s), 1278 (m), 1207 (m), 1145 (s), 1095 (m), 1074 (s), 1037 (s), 950 (w), 889 (w), 819 (m), 748 (w), 667 (w), 620 (w) cm^{-1}.

MS (ESI-pos.) *No MS signal for* **S3**-*iso was detected by ESI.*

3.3.5 Acetale Cleavage to iso-Resveratrone 2-*iso*

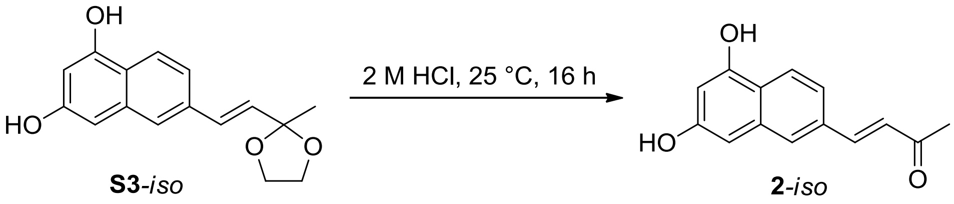

A round bottom flask was charged with acetale **S3**-*iso* (24.0 mg, 0.09 mmol) which was dissolved in THF (1 mL). HCl (0.70 mL, 2 M) was added to the solution and the reaction mixture was stirred overnight at rt. The mixture was diluted with Et_2O (30 mL) and then washed with Brine (2x10 mL) and aqueous, saturated NH_4Cl-solution (1x10 mL). The organic layer was dried over Na_2SO_4 and the solvent was removed *in vacuo*. The product **2**-*iso* (16 mg, 0.07 mmol, 78 %) was obtained as a yellow solid.

^{1}H-NMR (300 MHz, MeOH-d_4) δ = 8.06 (d, J = 8.8 Hz, 1 H), 7.68 – 7.77 (m, 2 H), 7.45 (dd, J = 8.8, 1.6 Hz, 1 H), 6.83 (d, J = 16.6 Hz, 1 H), 6.70 (d, J = 0.9 Hz, 1 H), 6.54 (d, J = 2.2 Hz, 1 H), 2.40 (s, 3 H) ppm.

^{13}C-NMR (75 MHz, MeOH-d_4): δ = 201.6, 158.0, 156.1, 146.6, 137.3, 134.0, 130.1, 127.7, 124.4, 122.5, 120.3, 103.3, 102.7, 27.5 ppm.

IR-FTR: $\tilde{\nu}$ = 3801 (w), 3748 (w), 3671 (w), 3648 (w), 3610 (w), 3565 (w), 3187 (m), 2919 (m), 2524 (m), 2360 (m), 2159 (s), 2028 (s), 1976 (s), 1670 (w), 1575 (s), 1519 (w), 1457 (w), 1398 (s), 1363 (m), 1263 (s), 1203 (m), 1178 (s), 1151 (s), 1076 (s), 1004 (m), 964 (m), 885 (w), 840 (w), 813 (m), 721 (w), 669 (m), 615 (m) cm^{-1}.

MS (ESI-pos.) *No MS signal for* **2**-*iso was detected by ESI.*

3.4 Synthesis of a clickable iso-Resveratrone Conjugate

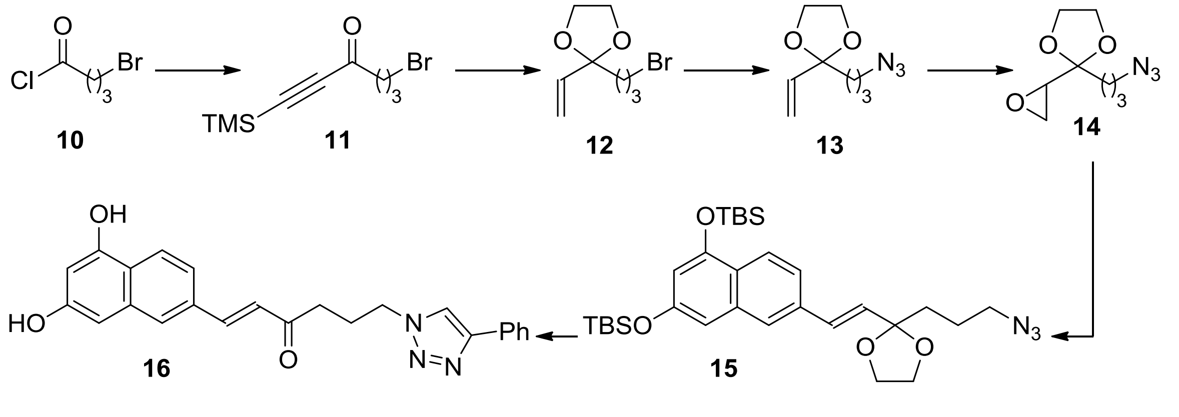

3.4.1 7-Bromo-1-(trimethylsilyl)hept-1-yn-3-one (11)

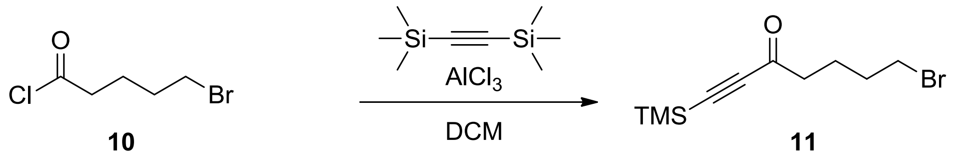

Under an atmosphere of argon aluminiumtrichloride (0.86 g, 6.52 mmol, 1.30 eq.) was dissolved in dry DCM (10 mL). The solution was cooled to 0°C. 5-Bromopentanoylchloride **10** (1.00 g, 5.01 mmol, 1.00 eq.) and bis(trimethylsilyl)acetylen (0.85 g, 5.01 mmol, 1.00 eq.) were dissolved in dry DCM (9 mL) and added dropwise to the aluminiumtrichloride solution at 0 °C over the course of 10 min. The cooling bath was removed and the reaction mixture was stirred at rt for 30 min. Then the reaction mixture was cooled to 0 °C again and aqueous HCL (1M, 9 mL) was added dropwise. The mixture was extracted with DCM (2x15 mL). All organic layers were collected, washed with brine (1x15 mL), dried with $MgSO_4$ and the solvent was evaporated under reduced pressure. The crude product was purified by column chromatography (SiO_2, CyHex/EtOAc, 94:6) to obtain **11** (1.10 g, 4.21 mmol, 83 %) as a yellow oil.

R_f: 0.49 (CyHex/EtOAc, 94:6).

^{1}H-NMR (400 MHz, $CDCl_3$): δ = 3.40 (t, *J* = 6.4 Hz, 2H), 2.59 (t, *J* = 7.0 Hz, 2H), 1.94 – 1.75 (m, 4H), 0.26 – 0.18 (m, 9H) ppm.

^{13}C-NMR (101 MHz, $CDCl_3$): δ = 187.1, 101.9, 98.4, 44.3, 33.1, 31.8, 22.5, 22.3, -0.7 ppm.

IR-FTR: ṽ = 2960, 2900, 2836, 2790, 2661, 2489, 2150, 2015, 1953, 1872, 1766, 1675, 1446, 1407,1353, 1251, 842, 761, 703, 620 cm^{-1}.

MS (ESI-pos.) *m/z* = calc. for $C_{10}H_{17}Br_1O_1Si$ $[M+H]^+$ 261.0305, found 261.0303.

3.4.2 2-(4-Bromobutyl)-2-vinyl-1,3-dioxolane (12)

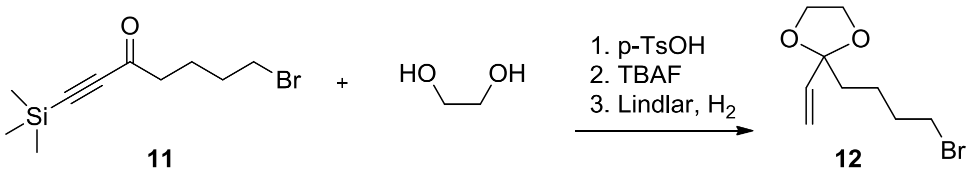

11 12

In a three-neck flask equipped with a Dean Stark apparatus compound **11** (1 g, 3.83 mmol, 1.00 eq.) was dissolved in benzene (25 mL) under an atmosphere of argon. Ethylenglycol (0.23 g, 3.83 mmol, 1.00 eq.) and *p*-TsOH (46.1 mg, 267 µmol, 0.07 eq.) were added, after which the mixture was heated to reflux for 16 h. The reaction mixture was cooled to rt and saturated aqueous $NaHCO_3$ (20 mL) was added. The aqueous layer was extracted with Et_2O (2x20 mL), the organic layers were collected, dried with $MgSO_4$ and concentrated *in vacuo*.

^{1}H NMR (300 MHz, $CDCl_3$) δ = 3.91 - 4.15 (m, 4 H), 3.43 (t, J=6.6 Hz, 2 H), 1.86 - 2.01 (m, 4 H), 1.65 - 1.79 (m, 2 H), 0.17 - 0.23 (m, 9 H) ppm. **^{1}H NMR data taken from the crude acetale**

The residue was dissolved in THF (10 mL) and cooled to 0 °C. A 1M solution of tetrabutylammoniumfluoride in THF (4.06 mL, 4.06 mmol, 1.06 eq.) was added and stirred for 1 h at rt. Subsequently brine (10 mL) was added and the mixture was extracted with Et_2O (3x20 mL), washed with saturated aqueous NH_4Cl (1x15 mL), dried with $MgSO_4$, and the solvent was evaporated under reduced pressure.

^{1}H-NMR (300 MHz, $CDCl_3$) δ = 3.93 - 4.22 (m, 4 H), 3.43 (t, J=6.7 Hz, 2 H), 2.52 (s, 1 H), 1.88 - 2.02 (m, 4 H), 1.66 - 1.79 (m, 2 H) ppm. **^{1}H NMR data taken from the crude desilylation product.**

The residue was dissolved in EtOAc (43 mL) and pyridine (0.15 ml, 1.91 mmol, 0.5 eq.) was added. Lindlar catalyst (100 mg, 5 % palladium over $CaCO_3$) was added and the reaction mixture was stirred for 16 h under an atmosphere of hydrogen. The catalyst was filtrated and the solvent evaporated under reduced pressure. The crude product was purified by silica gel column chromatography (CyHex/EtOAc/Et_3N 94:6:1) to obtain compound **12** (0.65 g, 2.76 mmol, 72 %) as colorless liquid.

R_f: 0.33 (CyHex/EtOAc/Et_3N 94:6:1).

^{1}H-NMR (400 MHz, $CDCl_3$): δ = 5.71 (dd, *J* = 17.2, 10.6 Hz, 1H), 5.35 (dd, *J* = 17.2, 1.8 Hz, 1H), 5.17 (dd, *J* = 10.6, 1.8 Hz, 1H), 3.98 – 3.83 (m, 4H), 3.39 (d, *J* = 6.8 Hz, 2H), 1.92 – 1.83 (m, 2H), 1.75 – 1.68 (m, 2H), 1.58 – 1.49 (m, 2H) ppm.

^{13}C-NMR (101 MHz, $CDCl_3$): δ = 137.6, 115.7, 108.9, 64.7, 37.2, 33.8, 33.0, 22.3 ppm.

IR-FTR: ṽ = 3089, 2950, 2884, 2682, 2292, 2051, 1976, 1870, 1677, 1641, 1457, 1432, 1403,1348,1295, 1267, 1211, 1191, 1110, 1041, 991, 935, 867, 802, 761, 740, 647 cm^{-1}.

MS (ESI-pos.) *m/z = Due to the low polarity of **12** no MS signal was detectable by ESI.*

3.4.3 2-(4-Azidobutyl)-2-vinyl-1,3-dioxolane (13)

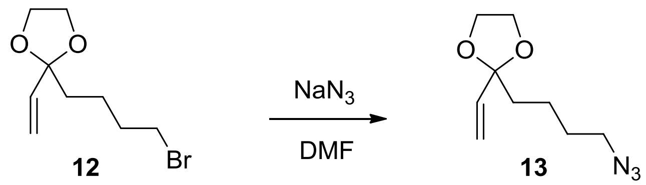

12 13

Bromide **12** (500 mg, 2.13 mmol, 1.00 eq.) was dissolved in DMF (6.75 mL) and sodium azide (415 mg, 6.38 mmol, 3.00 eq.) was added. The reaction mixture was stirred for 16 h at rt. Subsequently water (12.5 mL) was added at 0 °C and the mixture was extracted with Et_2O (3x25 mL). All organic layers were collected, dried with $MgSO_4$ and the solvent was evaporated under reduced pressure. The crude product was purified by column chromatography (SiO_2, CyHex/EtOAc/Et_3N 94:6:1) yielding azide **13** (410 mg, 2.07 mmol, 91 %) as colorless oil.

R_f: 0.23 (CyHex/EtOAc 94:6).

^{1}H-NMR (400 MHz, $CDCl_3$): δ = 5.71 (dd, *J* = 17.2, 10.6 Hz, 1H), 5.35 (dd, *J* = 17.2, 1.8 Hz, 1H), 5.17 (dd, *J* = 10.6, 1.8 Hz, 1H), 3.97 – 3.82 (m, 4H), 3.25 (t, *J* = 6.9 Hz, 2H), 1.75 – 1.69 (m, 2H), 1.66 – 1.57 (m, 2H), 1.52 – 1.42 (m, 2H) ppm.

^{13}C-NMR (101 MHz, $CDCl_3$): δ = 137.6, 115.7, 108.9, 64.7, 51.5, 37.6, 29.0, 20.8.

IR-FTR: ṽ = 3338, 3091, 2950, 2883, 2680, 2503, 2092, 1870, 1677, 1641, 1614, 1457, 1405, 1348, 1278, 1255, 1207, 1135, 1043, 993, 937, 871, 796, 715, 653, 617 cm^{-1}.

MS (ESI-pos.) *m/z = Due to the low polarity of **13** no MS signal was detectable by ESI.*

3.4.4 2-(4-Azidobutyl)-2-(oxiran-2-yl)-1,3-dioxolane (14)

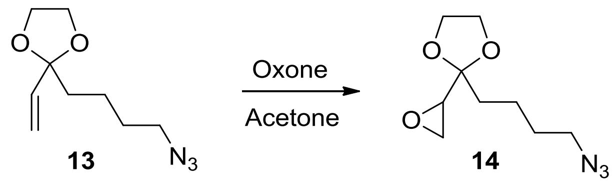

13 14

Azide **13** (200 mg, 1.01 mmol, 1.00 eq.) was dissolved in acetone (7.3 mL). The solution was cooled to 0 °C and $NaHCO_3$ (851 mg, 10.14 mmol, 10 eq.) was added. A solution of oxone (852 mg, 5.07 mmol, 5.00 eq.) in water (5 mL) was added dropwise to the reaction mixture, which was then stirred for 4 days at rt. The reaction mixture was filtered and the liquid phase was diluted with brine (15 mL) and extracted with Et_2O (3 x 15 mL). All organic layers were collected, dried with $MgSO_4$ and the solvent was evaporated under reduced pressure. The crude product was purified by column chromatography (SiO_2, CyHex/EtOAc/Et_3N 89:10:1) to obtain epoxide **14** (110 mg, 0.51 mmol, 51 %) as colorless oil.

R_f: 0.18 (CyHex/EtOAc 9:1).

^{1}H-NMR (400 MHz, $CDCl_3$): δ = 4.08 – 3.85 (m, 4H), 3.27 (t, *J* = 6.7 Hz, 2H), 3.01 (dd, *J* = 3.9, 2.8 Hz, 1H), 2.69 (qd, *J* = 5.7, 3.3 Hz, 2H), 1.77 – 1.71 (m, 2H), 1.66 – 1.57 (m, 2H), 1.56 – 1.46 (m, 2H) ppm.
^{13}C-NMR (101 MHz, $CDCl_3$): δ = 107.5, 66.4, 65.8, 54.7, 51.4, 43.7, 35.36, 29.0, 20.1 ppm.
IR-FTR: ṽ = 3338, 3064, 2952, 2894, 2686, 2508, 2094, 1714, 1637, 1616, 1521, 1457, 1403, 1348, 1265, 1213, 1182, 1128, 1078, 1035, 989, 948, 890, 823, 769, 738, 661, 615 cm^{-1}.
MS (ESI-pos.) *m/z* = calc. for $C_9H_{15}N_3O_3$ $[M+Na]^+$ 236.1006, found 236.1004.

3.4.5 Clickable *iso*-Resveratrone (15)

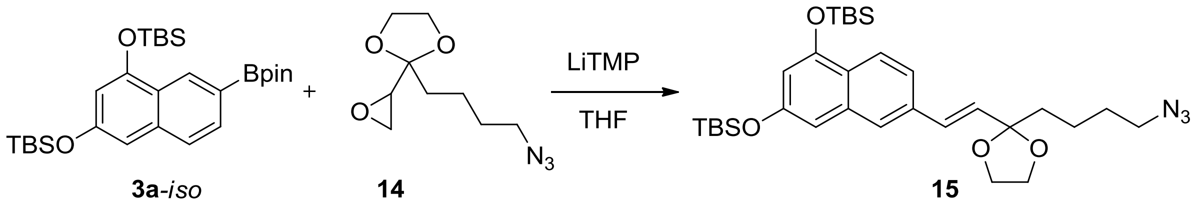

3a-*iso* **14** **15**

LiTMP was prepared under an atmosphere of argon by dropwise addition of *n*-BuLi in hexanes (18 µL, 187 µmol, 2.00 eq., 1.6 M) to 2,2,6,6-Tetramethylpiperidine (35 µL, 206 µmol, 2.20 eq.) in dry THF (0.5 mL) at 0 °C and stirring for 30 min at rt. In a separate Schlenk tube, under an atmosphere of argon, epoxid **14** (20 mg, 94 µmol, 1.00 eq.) and boronic acid ester **3a**-*iso* (73 mg, 141 µmol, 1.50 eq.) were dissolved in dry THF (0.5 mL), before the LiTMP solution was added dropwise at 0 °C. The reaction mixture stirred for 24 h at rt and subsequently for 2 h at 60 °C. After that brine (5 mL) and 1 M NaOH (5 mL) were added and extracted with Et_2O (3x10 mL). All organic layers were collected, dried over Na_2SO_4 and the solvent was evaporated under reduced pressure. The crude product was purified by column chromatography (SiO_2, CyHex/EtOAc 9:1) yielding **15** (28 mg, 48 µmol, 51 %) as yellow oil.
R_f: 0.56 (CyHex/EtOAc 9:1).
^{1}H-NMR (400 MHz, DMSO-d_6): δ = 8.09 – 8.03 (m, 1H), 7.76 (t, *J* = 2.8 Hz, 1H), 7.70 (dd, *J* = 14.3, 10.8 Hz, 1H), 7.52 – 7.47 (m, 1H), 6.88 – 6.79 (m, 2H,), 6.57 – 6.51 (m, 1H), 3.30 (dt, *J* = 24.7, 6.5 Hz, 2H), 2.82 – 2.70 (m, 2H), 1.91 – 1.49 (m, 8H), 1.09 (s, 9H), 1.01 (s, 9H), 0.29 (s, 6H), 0.25 (s, 6H) ppm.
^{13}C-NMR (101 MHz, DMSO-d_6): δ = 199.8, 154.6, 152.9, 143.1, 135.5, 132.8, 129.2, 126.2, 124.9, 123.6, 120.8, 110.7, 109.3, 63.8, 51.4, 40.3, 28.6, 25.9, 25.8, 25.4, 21.5, 18.6, 18.4, -4.1 ppm.
IR-FTR: ṽ = 3650, 3461, 2991, 2940, 2908, 2736, 2669, 2601, 2310, 2169, 2084, 1889, 1739, 1556, 1467, 1444, 1373, 1299, 1236, 1099, 1045, 1002, 939, 846, 784, 634, 607 cm^{-1}.
MS (ESI-pos.) *m/z* = calc. for $C_{31}H_{49}N_3O_4Si_2$ $[M+H]^+$ 584.3334, found 584.3353.

3.4.6 Click reaction with phenylacetylene and deprotection to 16a

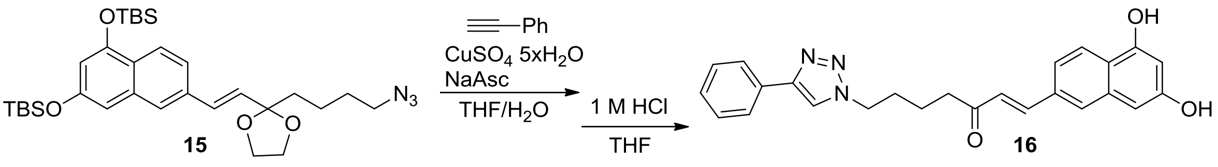

Under an atmosphere of argon azide **15** (10.0 mg, 17.1 µmol, 1.00 eq.) and phenylacetylen (1.92 mg, 18.8 µmol, 1.10 eq.) were dissolved in dry THF (2 mL). A mixture of copper(II)-sulfate-pentahydrat (0.42 mg, 1.71 µmol, 0.1 eq.) and sodium ascorbate (0.67 mg, 3.43 µmol, 0.2 eq.) were dissolved in degassed water (1 mL), added to the reaction mixture and stirred for 48 h at rt. The solvents were evaporated under reduced pressure and the residue was purified by swift column chromatography (SiO_2, CyHex/EtOAc 1:1) yielding the protected click product (2.5 mg, 3.64 µmol, 21 %) as a white solid, which was immediately used for the next reaction step.* **R_f:** 0.59 (CyHex/EtOAc 1:1). **MS (ESI-pos.)** *m/z* = calc. for $C_{39}H_{55}N_3O_4Si_2$ $[M+H]^+$ 686.3804, found 686.3801. The protected phenyl *iso*-resveratron **13a** (2.00 mg, 2.92 µmol, 1 eq.) was dissolved in dry THF (0.5 mL), to which 1 M aqueous HCl (0.5 mL) was added and the reaction mixture was stirred for 16 h at rt. Subsequently, Et_2O (10 mL) was added and the mixture was washed with brine (2x10 mL) and saturated aqueous NH_4Cl (2x10 mL). The organic layer was dried with Na_2SO_4, the solvent was evaporated under reduced pressure and the phenyl clicked *iso*-resveratron **16** (1.00 mg, 2.42 µmol, 82 %) was obtained as white solid.

^{1}H-NMR (600 MHz, MeOH-*d_4*): δ = 8.34 (d, *J* = 4.9 Hz, 1H), 8.04 (d, *J* = 8.6 Hz, 1H), 7.82 – 7.79 (m, 2H), 7.72 (d, *J* = 15.6 Hz, 2H), 7.45 (dd, *J* = 8.8, 1.5 Hz, 1H), 7.44 – 7.39 (m, 2H), 7.35 – 7.31 (m, 1H), 6.88 (d, J = 16.3 Hz, 1H), 6.68 – 6.62 (m, 1H), 6.51 (dd, *J* = 14.4, 2.0 Hz, 1H), 4.43 (dt, *J* = 94.9, 7.1 Hz, 1H), 2.83 (t, *J* = 7.3 Hz, 2H), 2.57 (t, *J* = 7.2 Hz, 1H), 2.07 – 2.01 (m, 2H), 1.90 – 1.81 (m, 1H), 1.73 (dt, *J* = 14.8, 7.4 Hz, 2H), 1.61 (dt, *J* = 14.8, 7.4 Hz, 1H) ppm.

^{13}C-NMR (151 MHz, MeOH-*d_4*): δ = 205.5, 202.6, 157.8, 155.9, 148.9, 145.3, 141.6, 137.2, 133.9, 131.7, 130.1, 129.9, 129.7, 129.3, 129.3, 128.6, 126.7, 126.6, 124.2, 123.3, 123.3, 122.7, 122.2, 122.1, 120.1, 103.1, 102.6, 102.5, 102.1, 54.8, 51.3, 51.2, 49.8, 43.2, 40.4, 30.6, 30.5, 22.2, 22.0 ppm.

IR-FTR: $\tilde{v}$ = 3135, 2923, 2848, 1671, 1627, 1583, 1525, 1459, 1400, 1284, 1232, 1170, 1147, 1108, 1078, 1002, 977, 892, 817, 763, 692, 671, 624 cm^{-1}.

MS (ESI-pos.) *m/z* = calc. for $C_{25}H_{23}N_3O_3$ $[M+H]^+$ 414.1812, found 414.1809.

***Comment:** Under these conditions partial deprotection occurred. While those side products might be salvageable by global deprotection, water free click conditions might be preferable.[3]

[3] S. Kovács, K. Zih-Perényi, Á. Révész, Z. Novák, *Synthesis* **2012**, *44*, 3722-3730.

4 NMR-Spectra

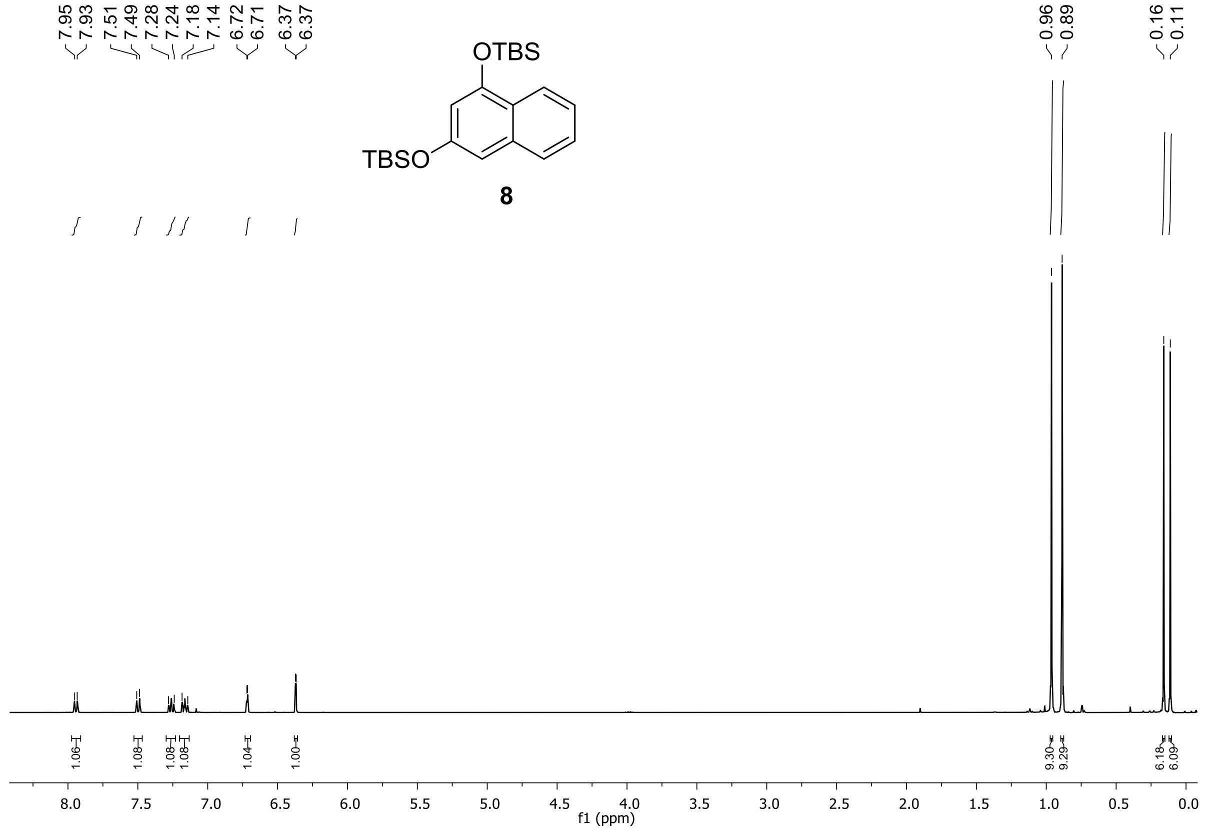

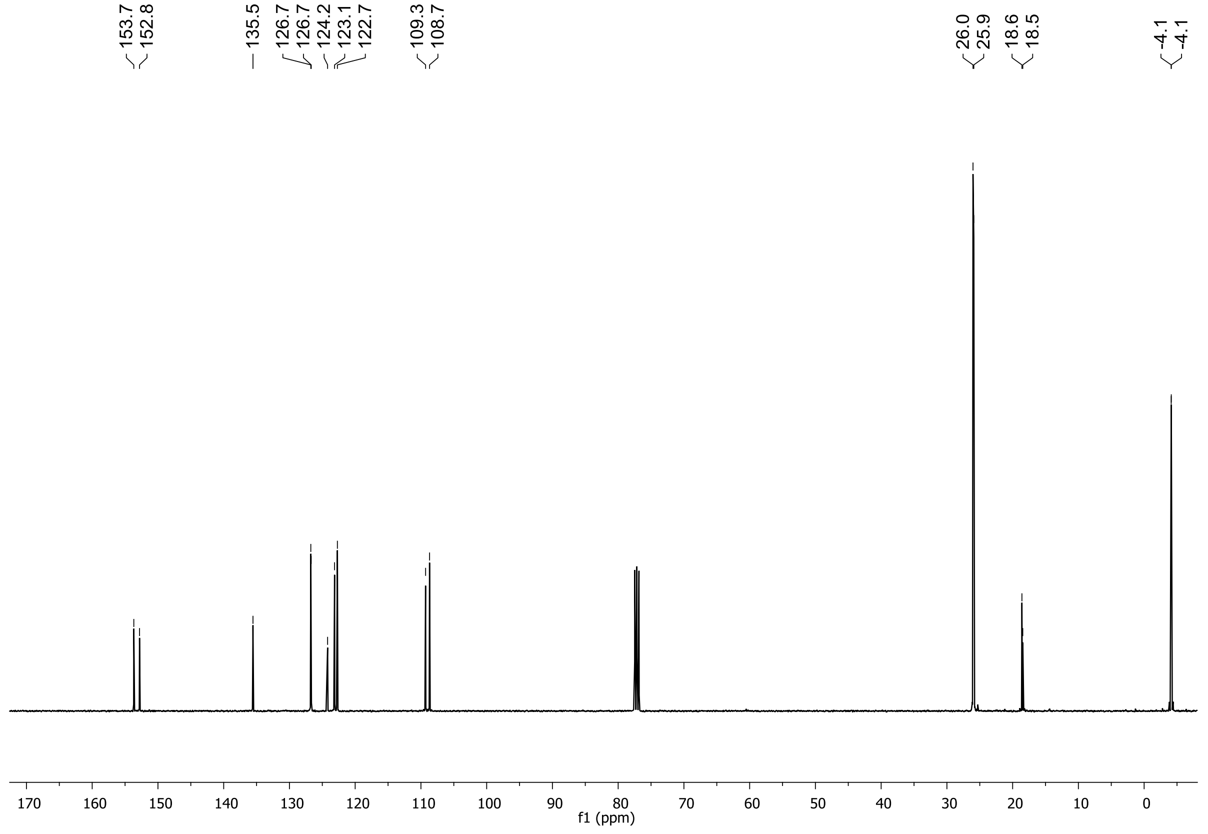

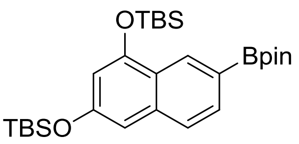

OTBS
Bpin
TBSO

3a

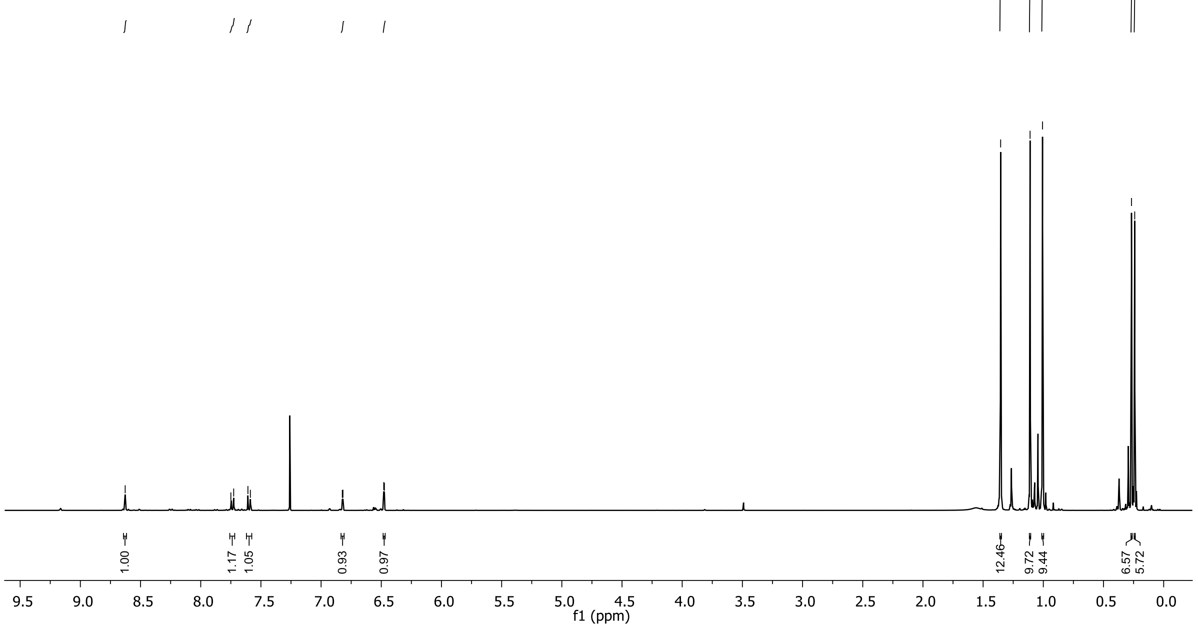

8.63
7.75
7.73
7.61
7.59
6.82
6.82
6.48
6.47
1.35
1.11
1.01
0.27
0.24
1.00
1.17
1.05
0.93
0.97
12.46
9.72
9.44
6.57
5.72
9.5 9.0 8.5 8.0 7.5 7.0 6.5 6.0 5.5 5.0 4.5 4.0 3.5 3.0 2.5 2.0 1.5 1.0 0.5 0.0
f1 (ppm)

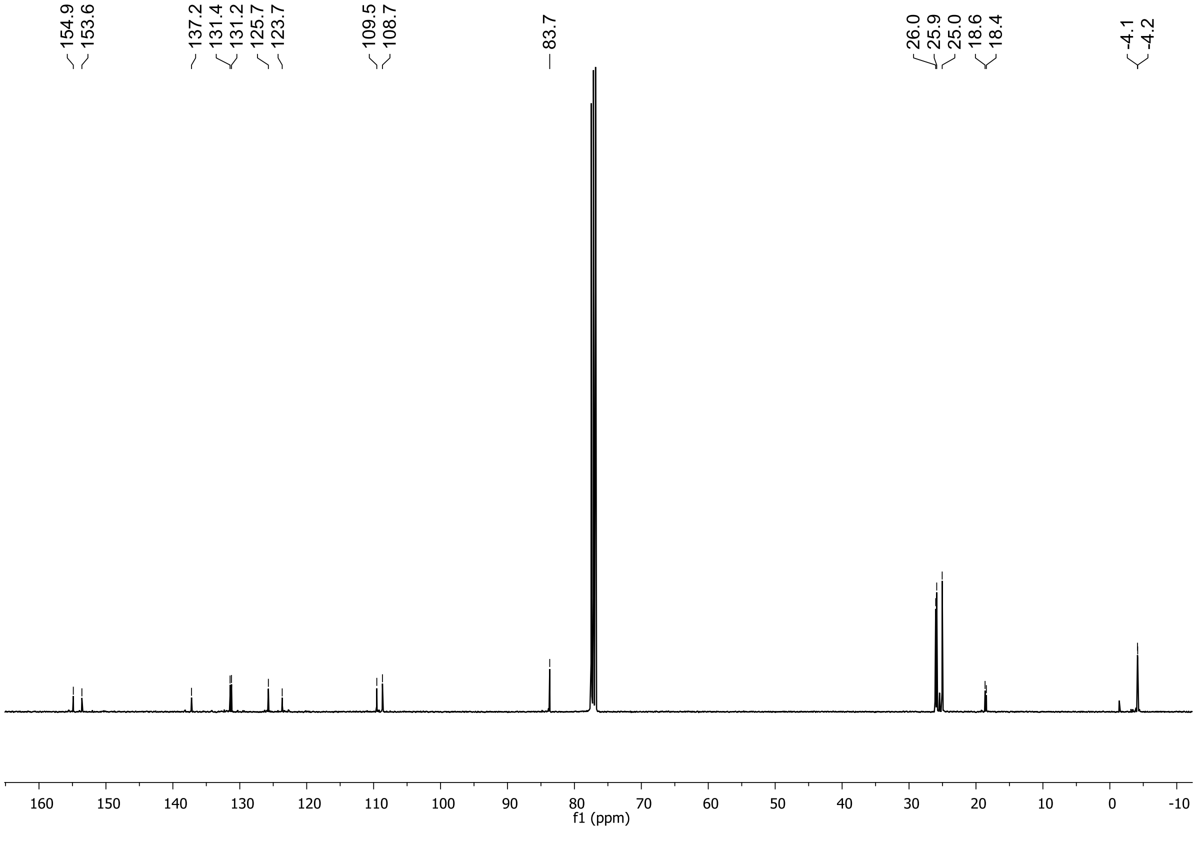

154.9
153.6
137.2
131.4
131.2
125.7
123.7
109.5
108.7
83.7
26.0
25.9
25.0
18.6
18.4
-4.1
-4.2
160 150 140 130 120 110 100 90 80 70 60 50 40 30 20 10 0 -10
f1 (ppm)

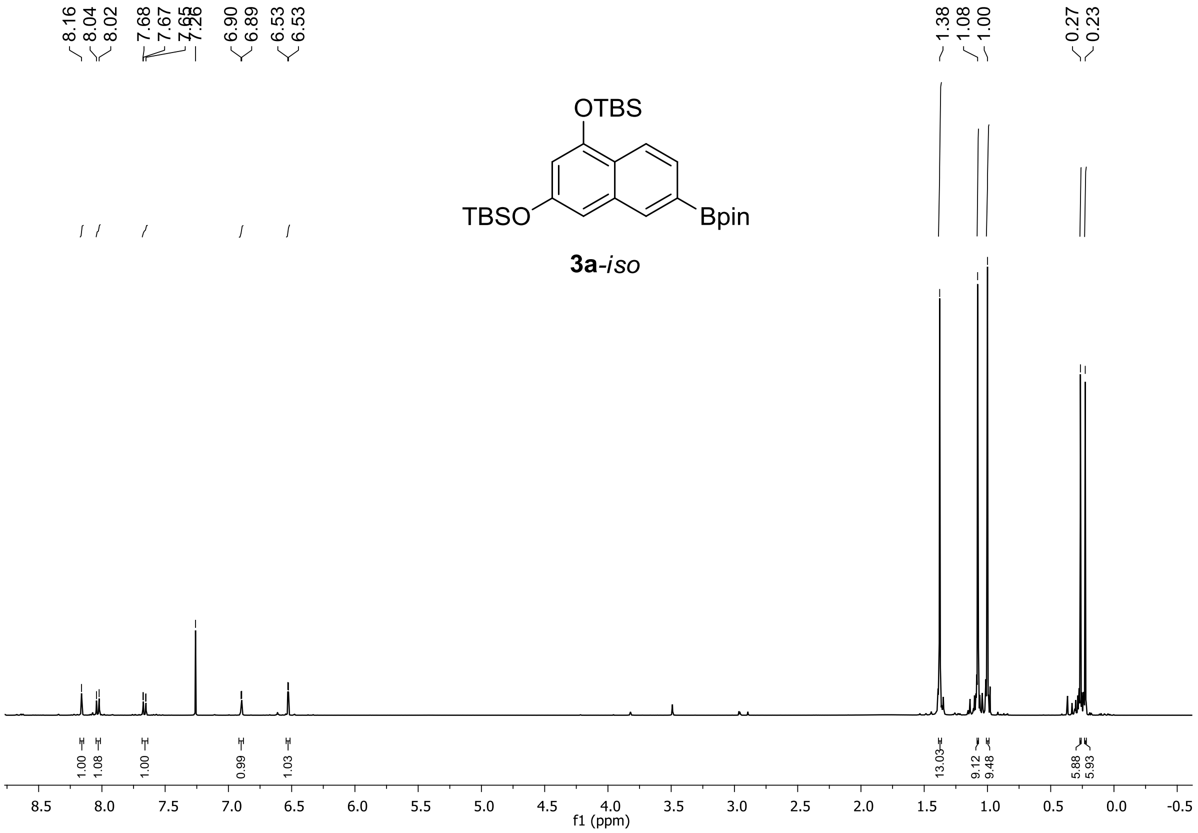
8.16
8.04
8.02
7.68
7.67
7.65
7.26
6.90
6.89
6.53
6.53
OTBS
TBSO
Bpin
1.38
1.08
1.00
0.27
0.23
1.00
1.08
1.00
0.99
1.03
13.03
9.12
9.48
5.88
5.93
8.5 8.0 7.5 7.0 6.5 6.0 5.5 5.0 4.5 4.0 3.5 3.0 2.5 2.0 1.5 1.0 0.5 0.0 -0.5
f1 (ppm)

3a-*iso*

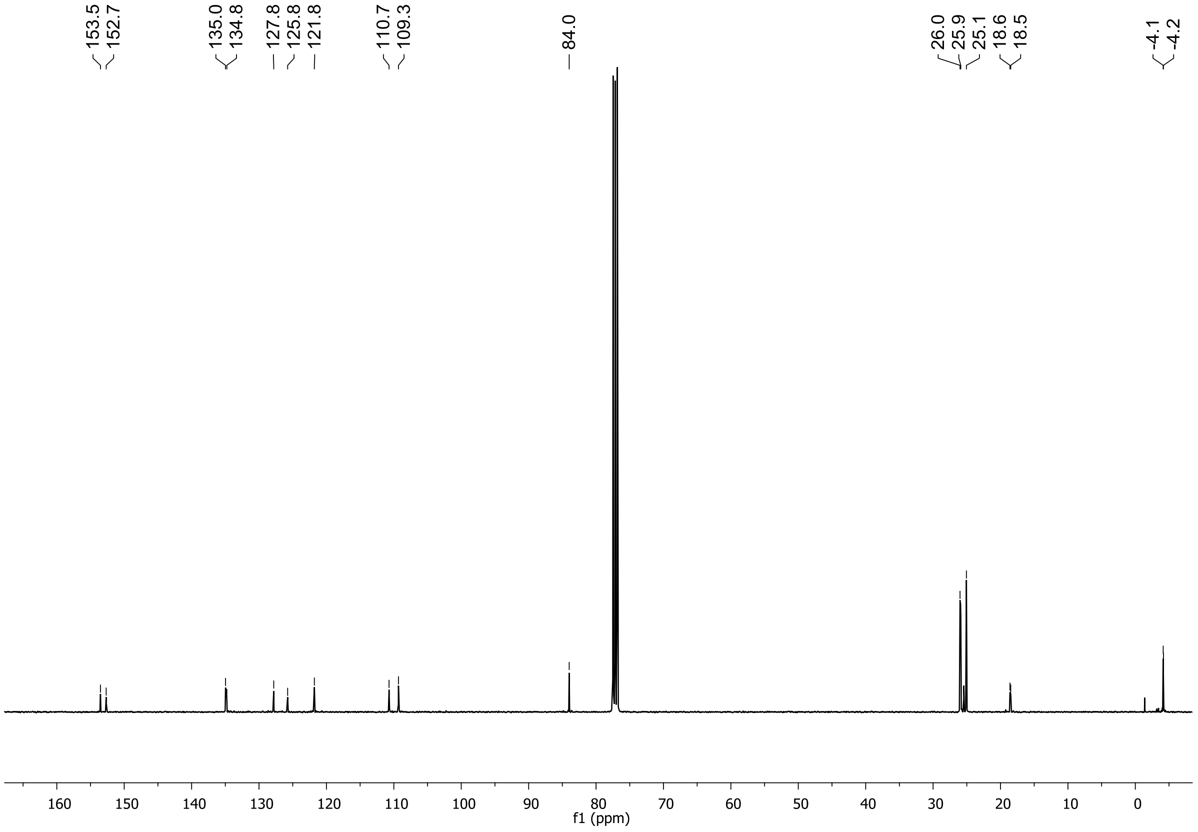
153.5
152.7
135.0
134.8
127.8
125.8
121.8
110.7
109.3
84.0
26.0
25.9
25.1
18.6
18.5
-4.1
-4.2
160 150 140 130 120 110 100 90 80 70 60 50 40 30 20 10 0
f1 (ppm)

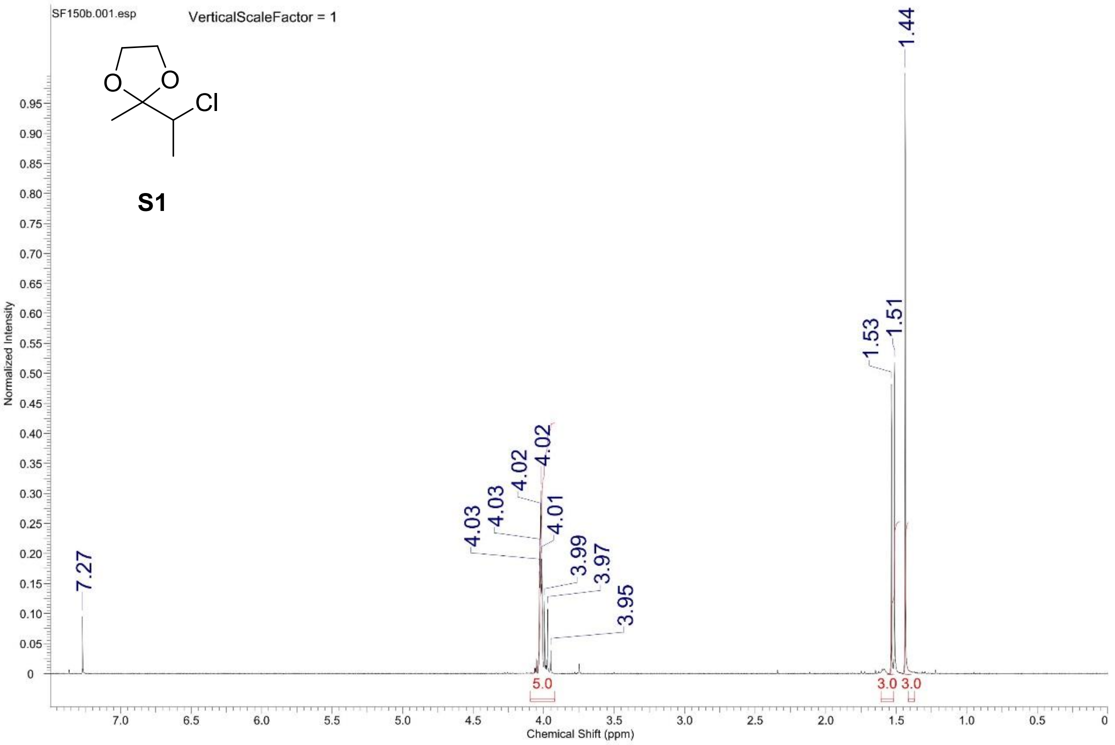
SF150b.001.esp
VerticalScaleFactor = 1
S1
7.27
4.03
4.03
4.02
4.02
4.01
3.99
3.97
3.95
1.53
1.51
1.44
5.0
3.0
3.0
Normalized Intensity
Chemical Shift (ppm)

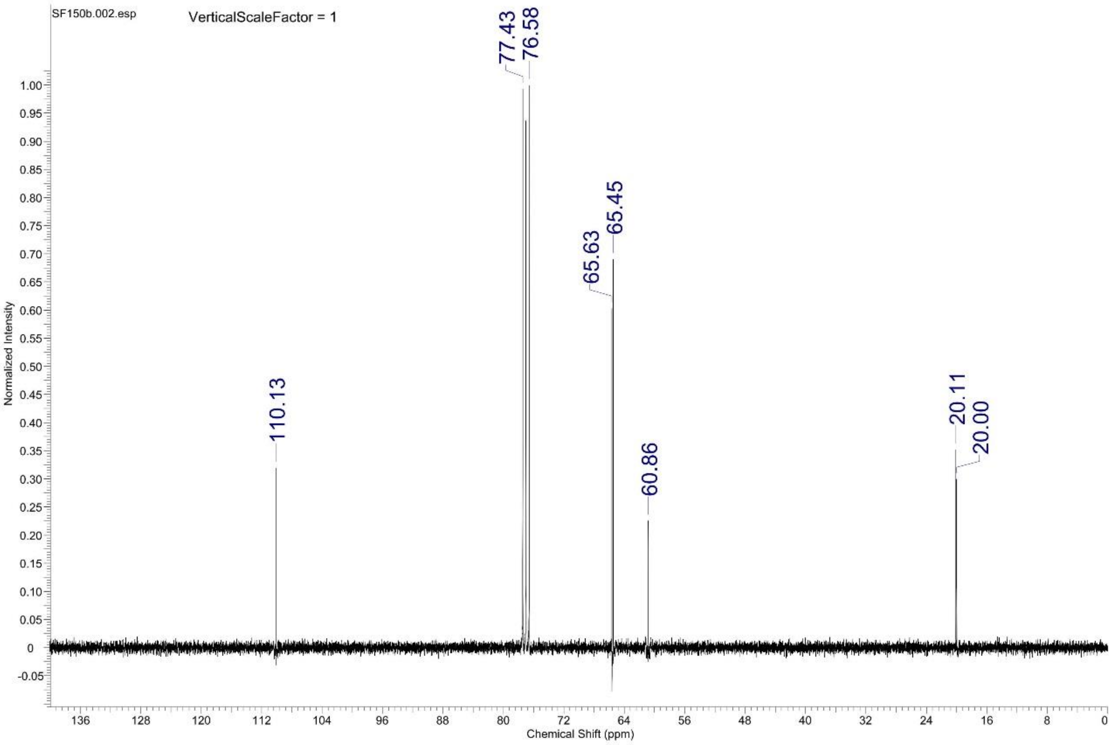
SF150b.002.esp
VerticalScaleFactor = 1
110.13
77.43
76.58
65.63
65.45
60.86
20.11
20.00
Normalized Intensity
Chemical Shift (ppm)

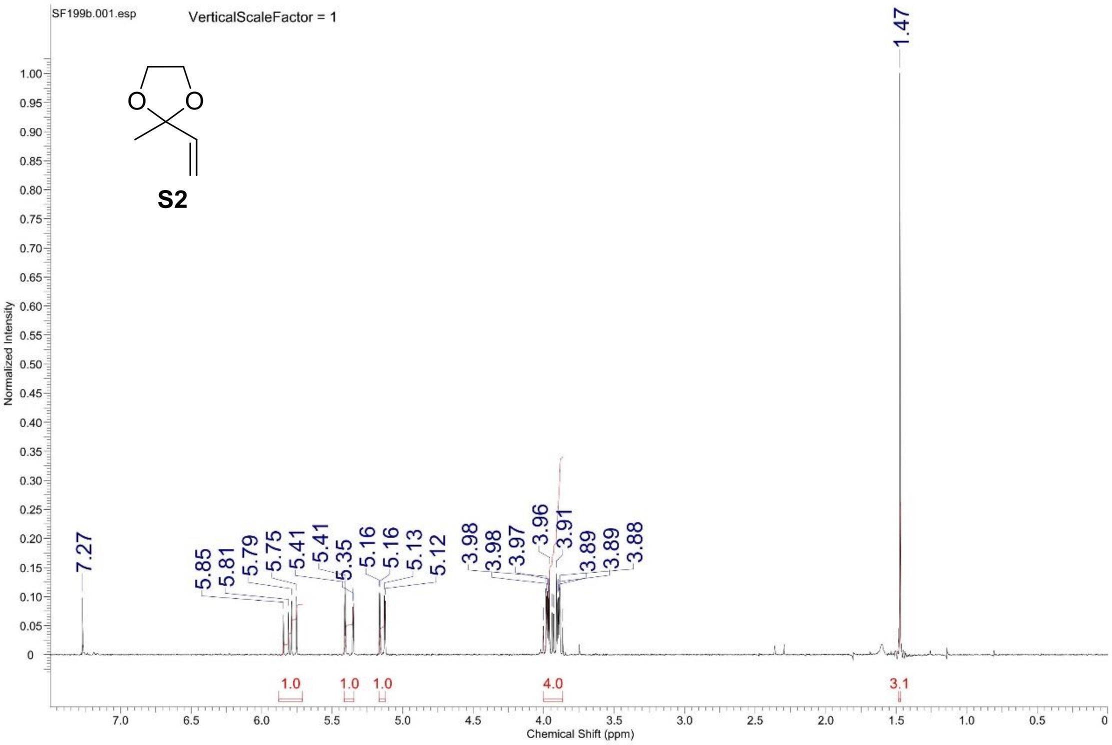
SF199b.001.esp
VerticalScaleFactor = 1
S2
O
O
Normalized Intensity
Chemical Shift (ppm)
7.27
5.85
5.81
5.79
5.75
5.41
5.41
5.35
5.16
5.16
5.13
5.12
3.98
3.98
3.97
3.96
3.91
3.89
3.89
3.88
1.47
1.0
1.0
1.0
4.0
3.1

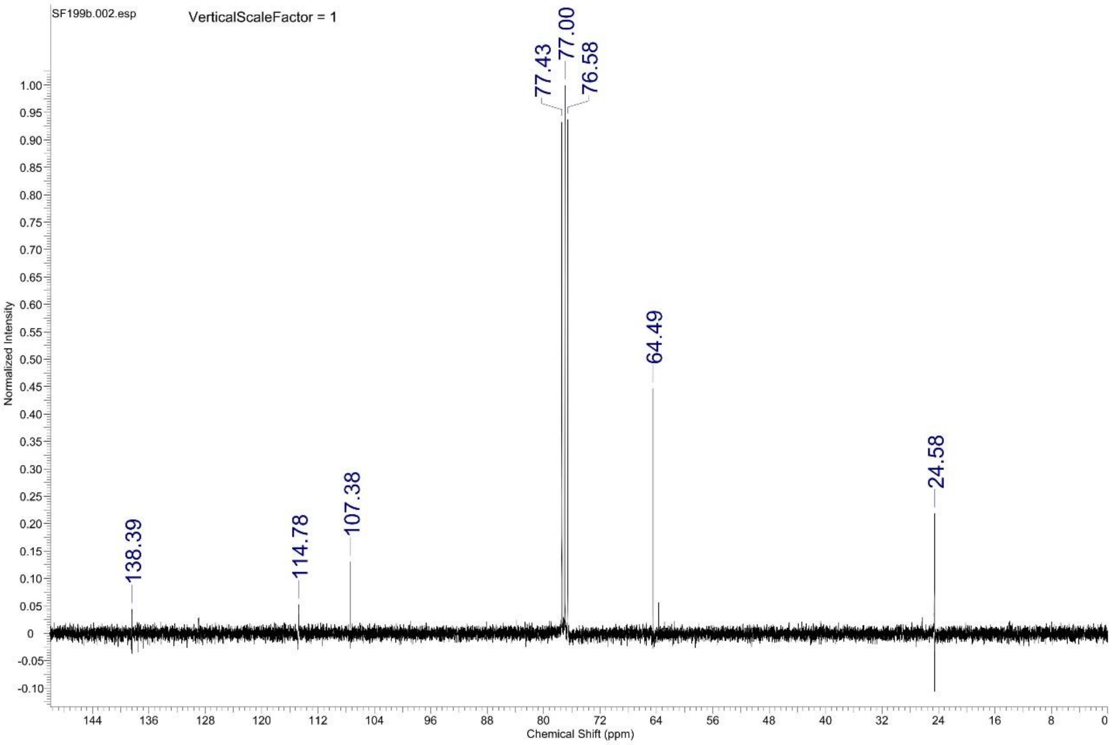
SF199b.002.esp
VerticalScaleFactor = 1
Normalized Intensity
Chemical Shift (ppm)
138.39
114.78
107.38
77.43
77.00
76.58
64.49
24.58

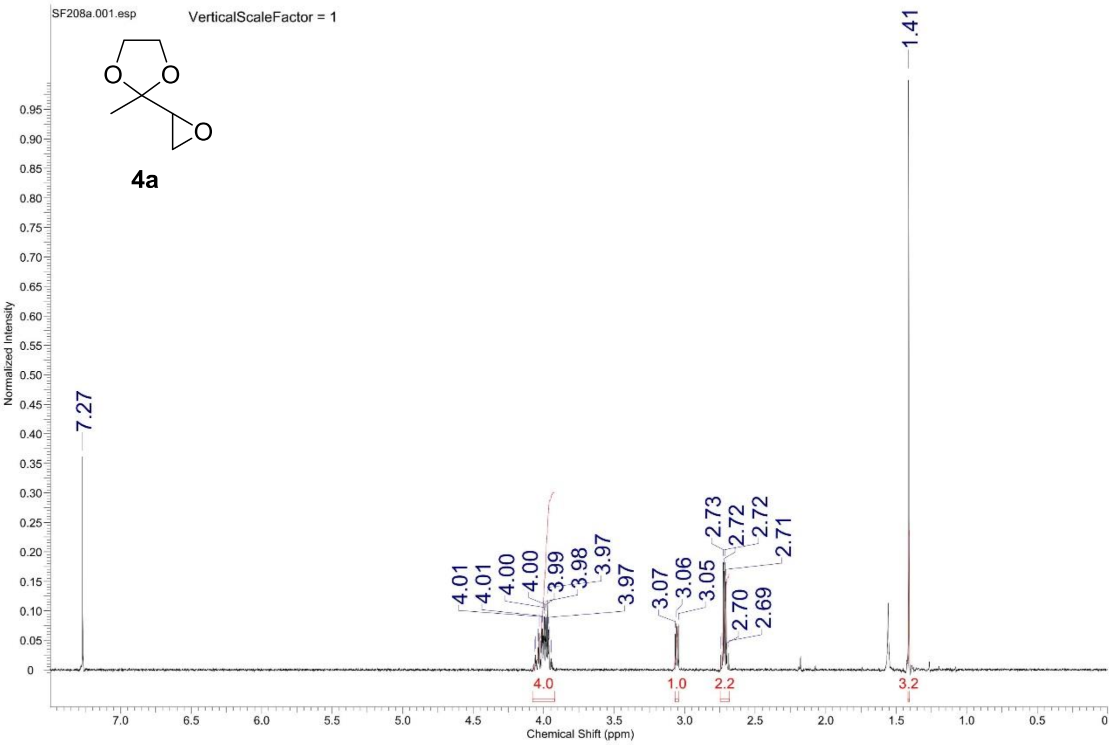
SF208a.001.esp
VerticalScaleFactor = 1
4a
Normalized Intensity
Chemical Shift (ppm)
7.27
4.01
4.01
4.00
4.00
3.99
3.98
3.97
3.97
3.07
3.06
3.05
2.73
2.72
2.72
2.71
2.70
2.69
1.41
4.0
1.0
2.2
3.2

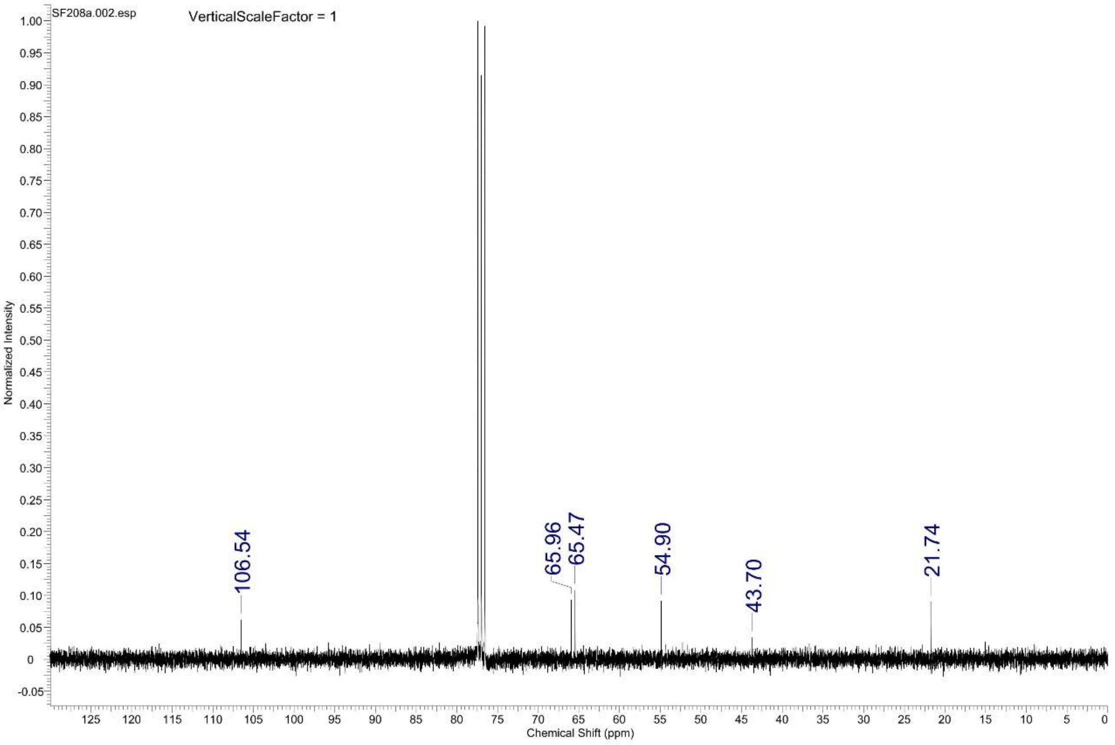
SF208a.002.esp
VerticalScaleFactor = 1
Normalized Intensity
Chemical Shift (ppm)
106.54
65.96
65.47
54.90
43.70
21.74

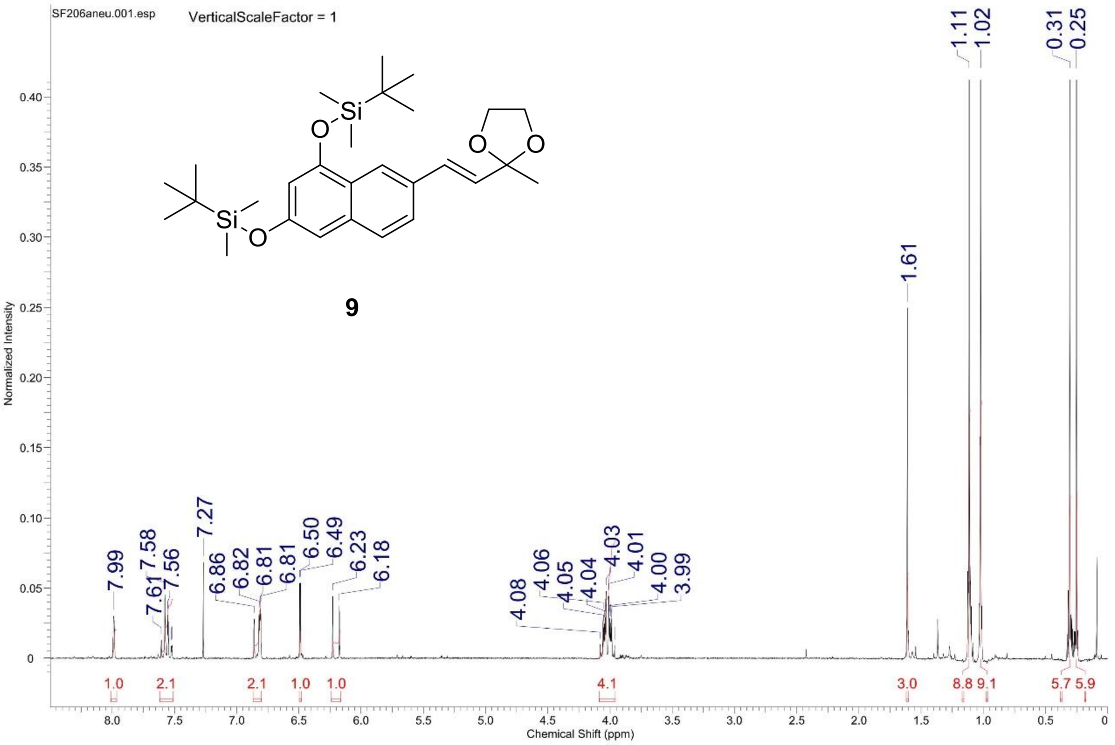
SF206aneu.001.esp
VerticalScaleFactor = 1
Si
O
O
Si
O
O
9
Normalized Intensity
Chemical Shift (ppm)
7.99 7.61 7.58 7.56 7.27 6.86 6.82 6.81 6.81 6.50 6.49 6.23 6.18
4.08 4.06 4.05 4.04 4.03 4.01 4.00 3.99
1.61 1.11 1.02 0.31 0.25
1.0 2.1 2.1 1.0 1.0 4.1 3.0 8.8 9.1 5.7 5.9

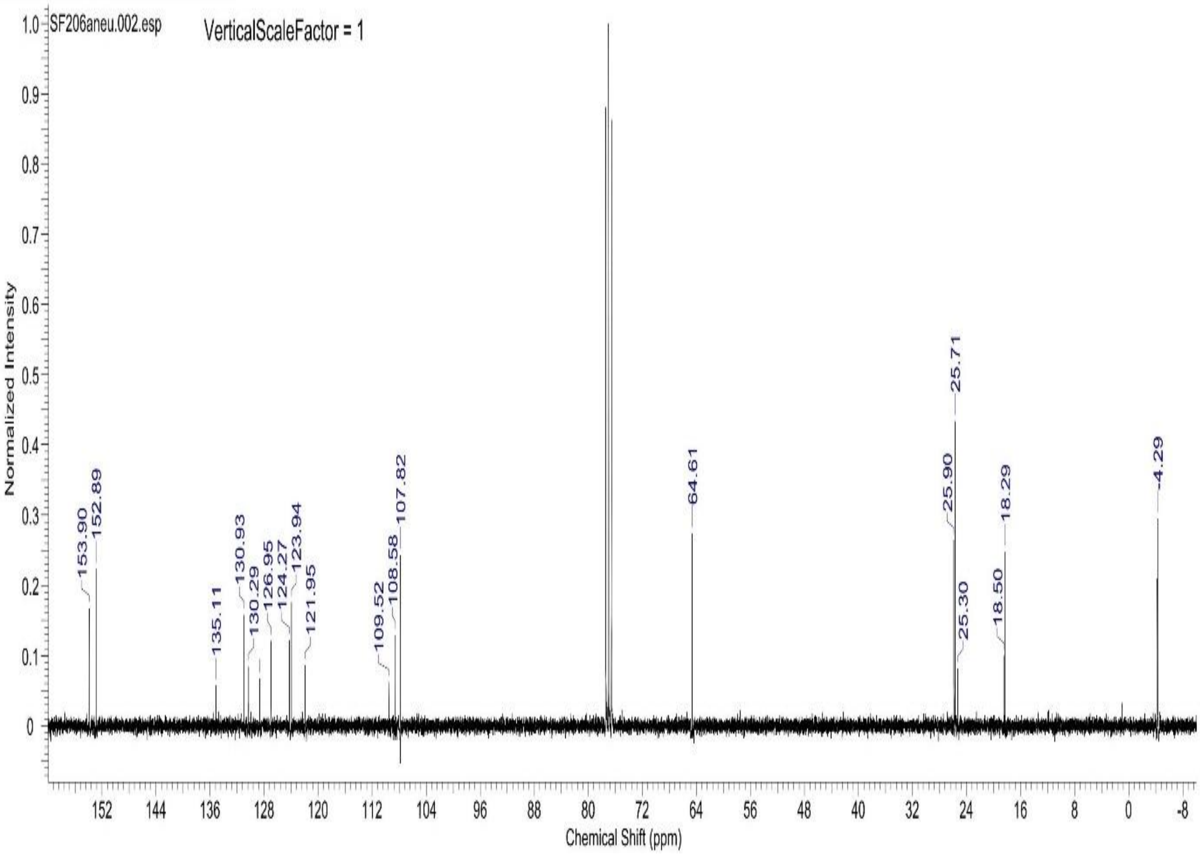
SF206aneu.002.esp
VerticalScaleFactor = 1
Normalized Intensity
Chemical Shift (ppm)
153.90 152.89 135.11 130.93 130.29 126.95 124.27 123.94 121.95 109.52 108.58 107.82 64.61 25.90 25.71 25.30 18.50 18.29 -4.29

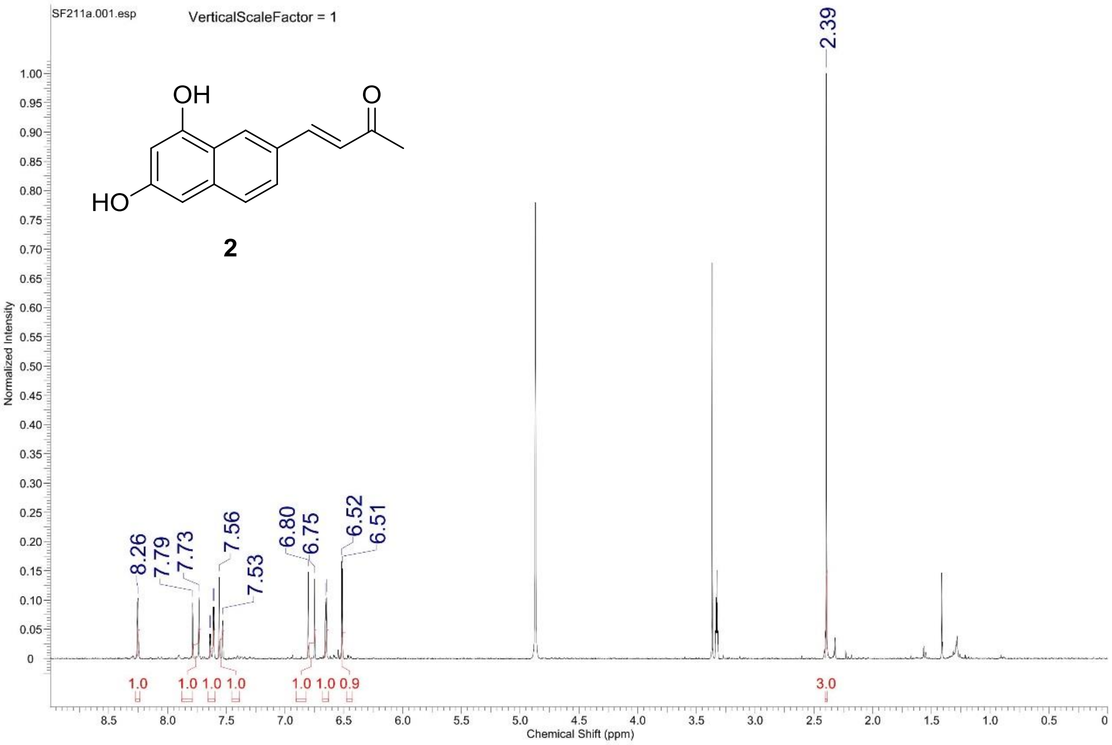
SF211a.001.esp
VerticalScaleFactor = 1
OH
O
HO
2
Normalized Intensity
Chemical Shift (ppm)
8.26
7.79
7.73
7.56
7.53
6.80
6.75
6.52
6.51
2.39
1.0
1.0
1.0
1.0
1.0
1.0
0.9
3.0

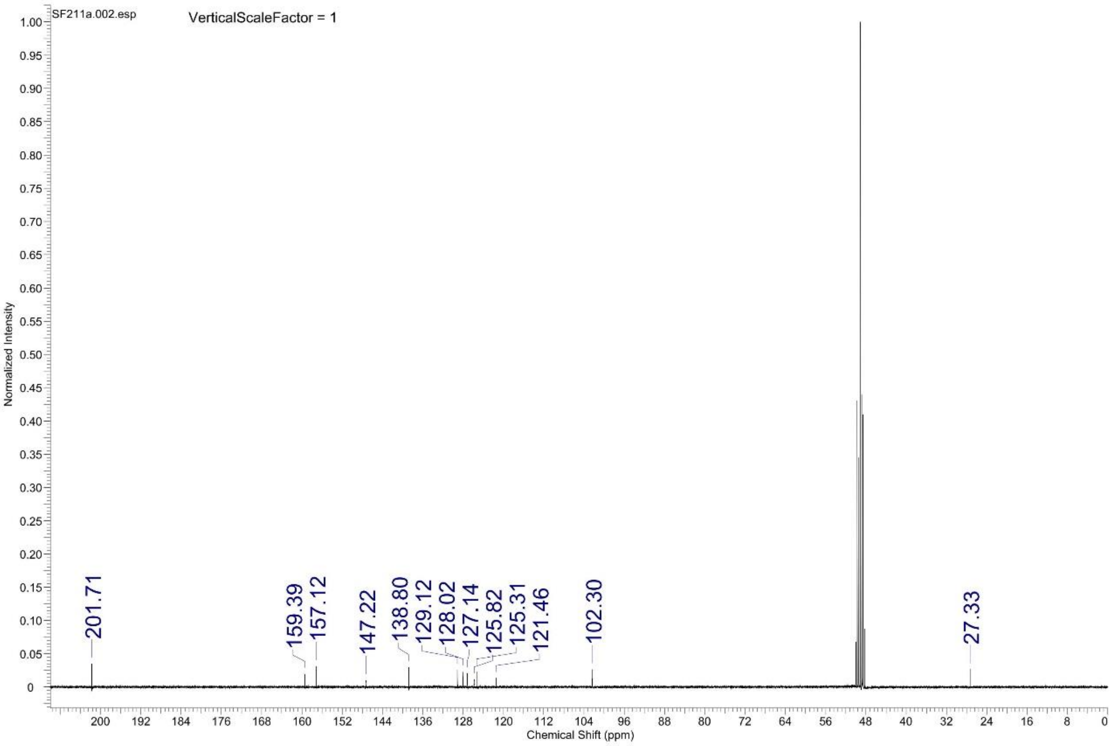
SF211a.002.esp
VerticalScaleFactor = 1
Normalized Intensity
Chemical Shift (ppm)
201.71
159.39
157.12
147.22
138.80
129.12
128.02
127.14
125.82
125.31
121.46
102.30
27.33

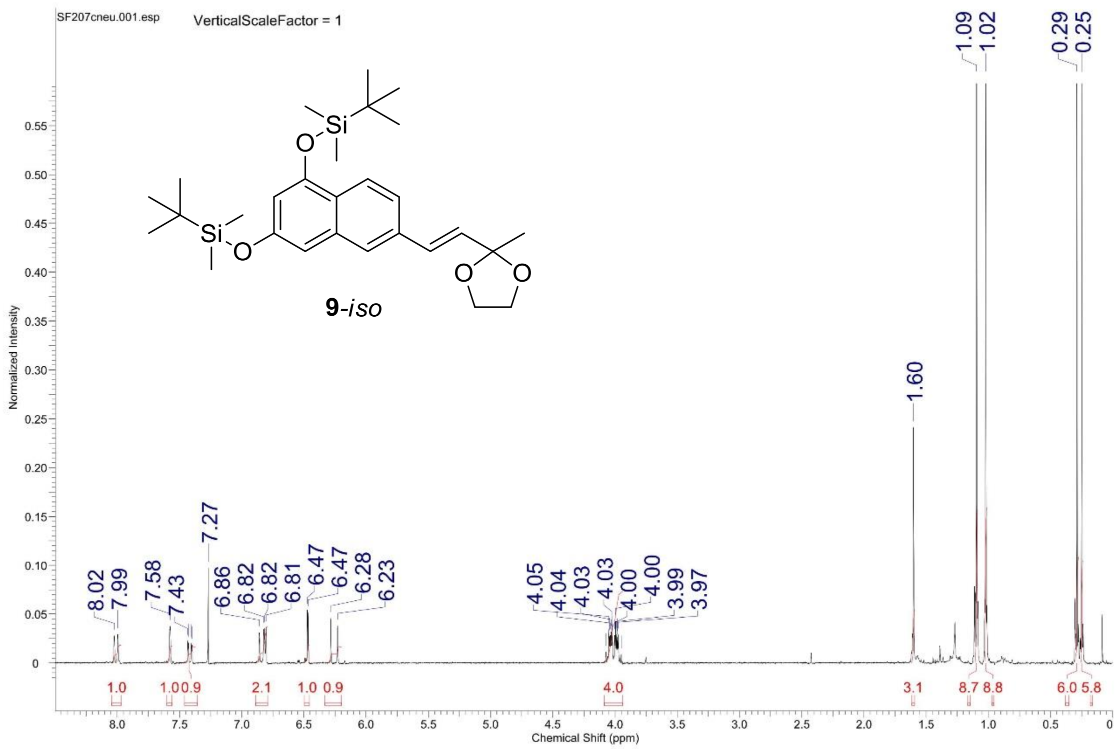
SF207cneu.001.esp
VerticalScaleFactor = 1
9-iso
Normalized Intensity
Chemical Shift (ppm)
8.02
7.99
7.58
7.43
7.27
6.86
6.82
6.82
6.81
6.47
6.47
6.28
6.23
4.05
4.04
4.03
4.03
4.00
4.00
3.99
3.97
1.60
1.09
1.02
0.29
0.25
1.0
1.00
0.9
2.1
1.0
0.9
4.0
3.1
8.7
8.8
6.0
5.8

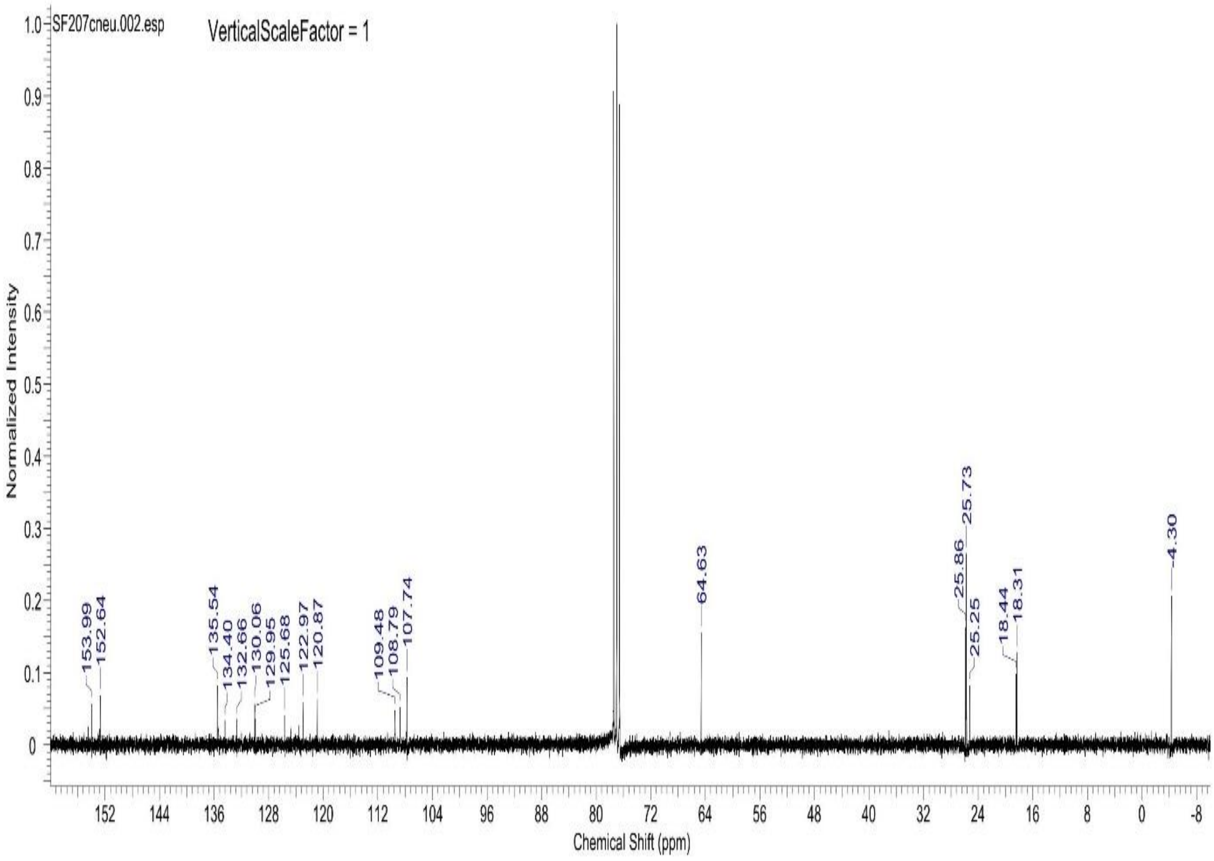
SF207cneu.002.esp
VerticalScaleFactor = 1
Normalized Intensity
Chemical Shift (ppm)
153.99
152.64
135.54
134.40
132.66
130.06
129.95
125.68
122.97
120.87
109.48
108.79
107.74
64.63
25.86
25.73
25.25
18.44
18.31
-4.30

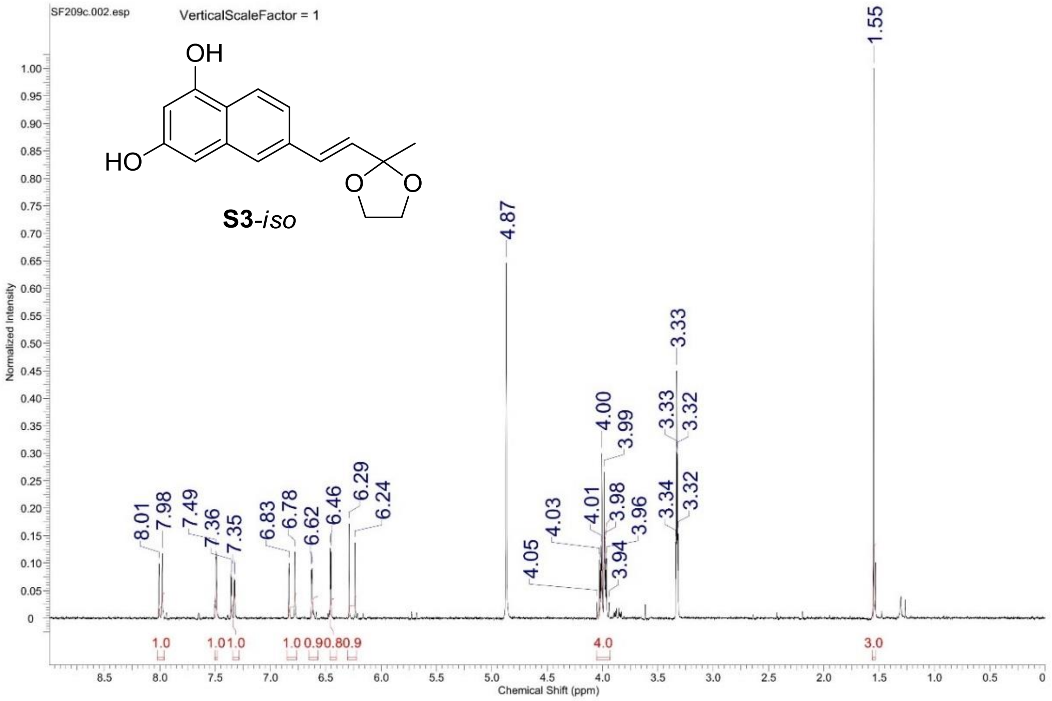
SF209c.002.esp
VerticalScaleFactor = 1
OH
HO
O
O
S3-iso
1.55
4.87
3.33
4.00
3.99
3.33
3.32
8.01
7.98
7.49
7.36
7.35
6.83
6.78
6.62
6.46
6.29
6.24
4.05
4.03
4.01
3.98
3.96
3.94
3.34
3.32
1.0
1.0
1.0
1.0
0.9
0.8
0.9
4.0
3.0
Normalized Intensity
Chemical Shift (ppm)

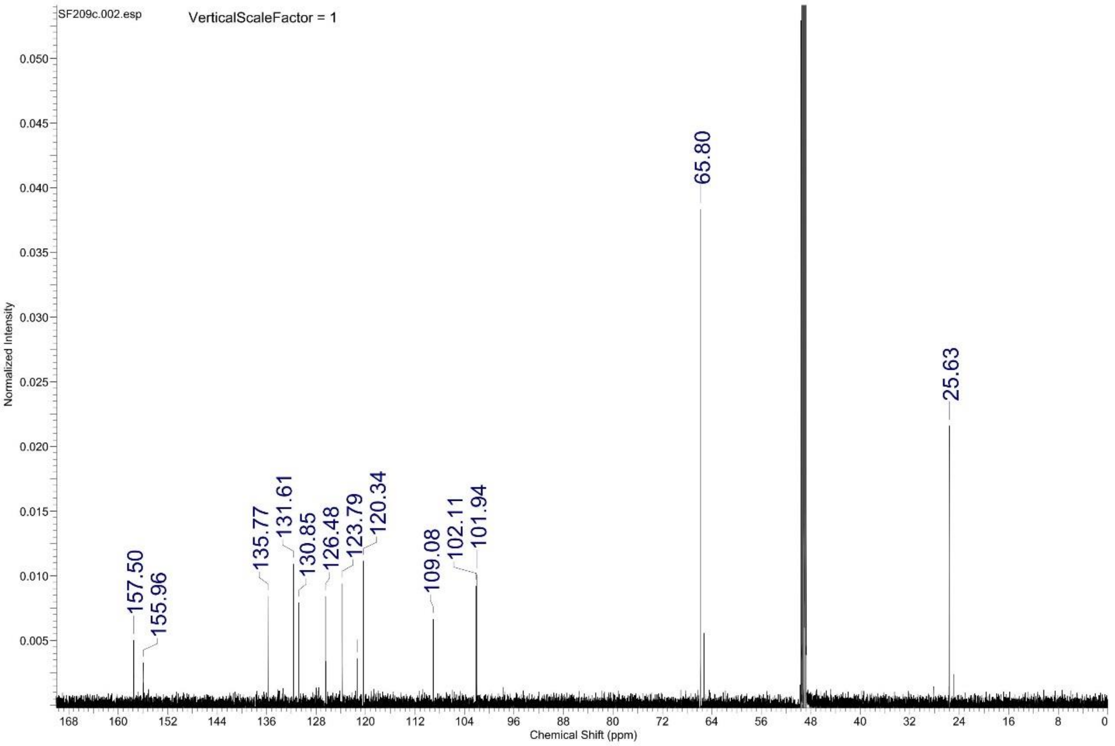
SF209c.002.esp
VerticalScaleFactor = 1
65.80
25.63
157.50
155.96
135.77
131.61
130.85
126.48
123.79
120.34
109.08
102.11
101.94
Normalized Intensity
Chemical Shift (ppm)

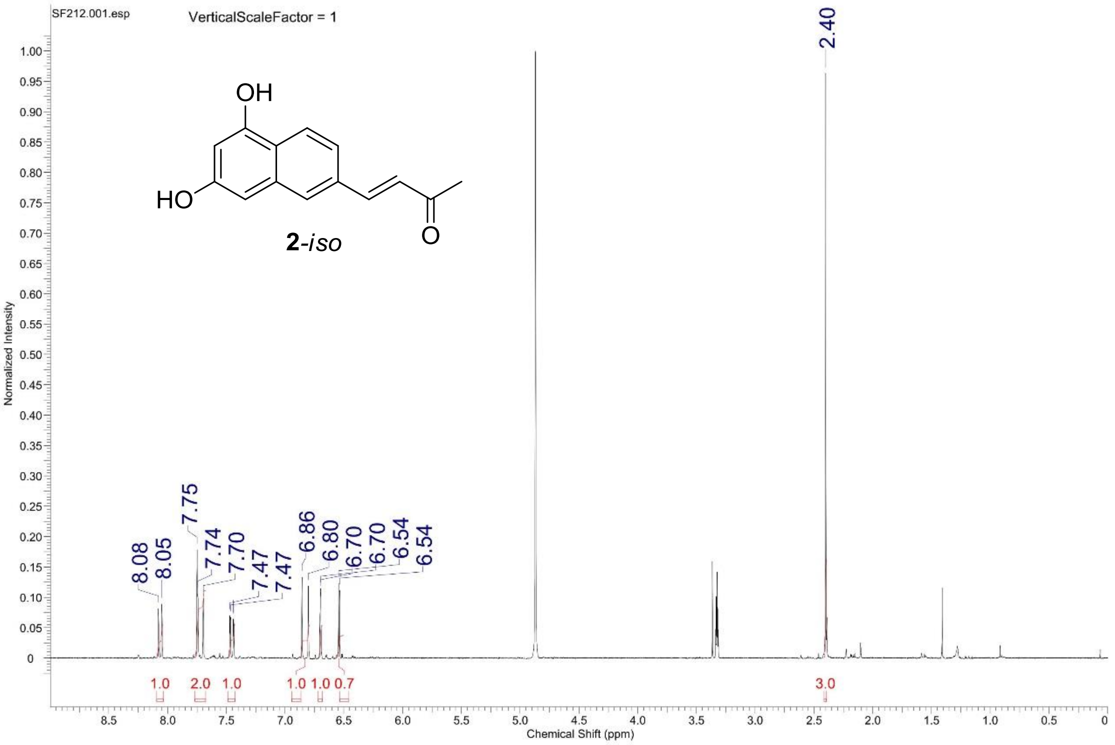
SF212.001.esp
VerticalScaleFactor = 1
OH
HO
2-*iso*
O
8.08
8.05
7.75
7.74
7.70
7.47
7.47
6.86
6.80
6.70
6.70
6.54
6.54
2.40
1.0
2.0
1.0
1.0
1.0
0.7
3.0
Normalized Intensity
Chemical Shift (ppm)

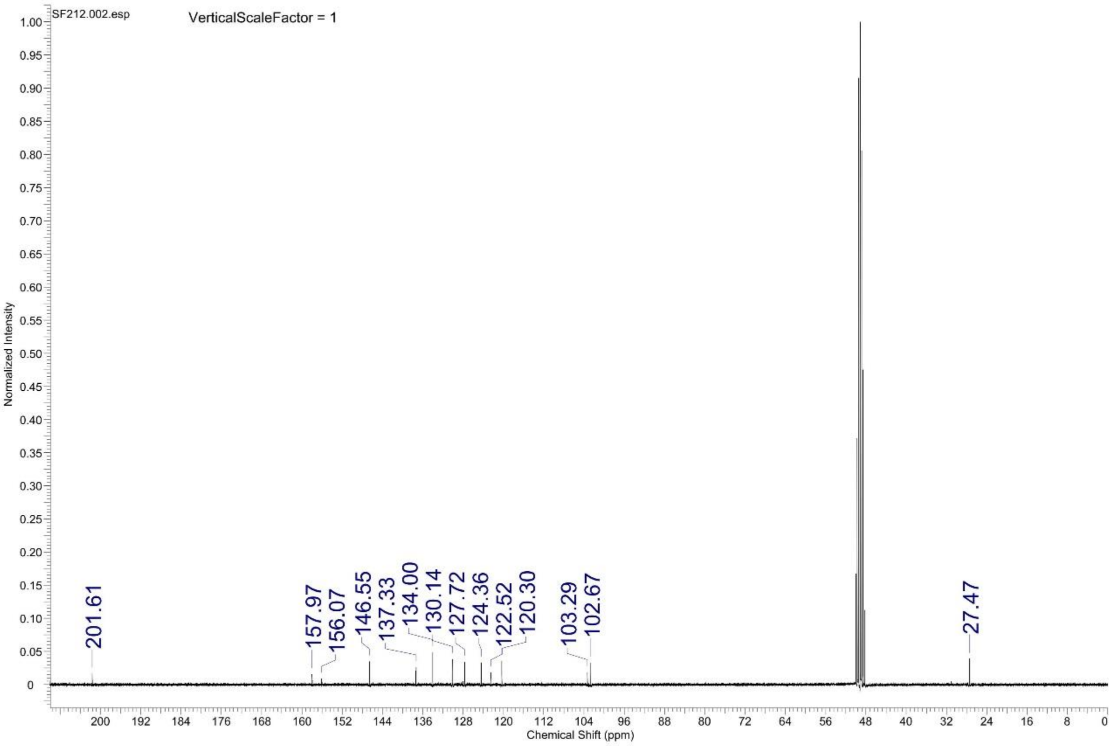
SF212.002.esp
VerticalScaleFactor = 1
201.61
157.97
156.07
146.55
137.33
134.00
130.14
127.72
124.36
122.52
120.30
103.29
102.67
27.47
Normalized Intensity
Chemical Shift (ppm)

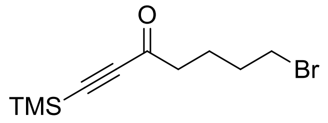
O
Br
TMS

11

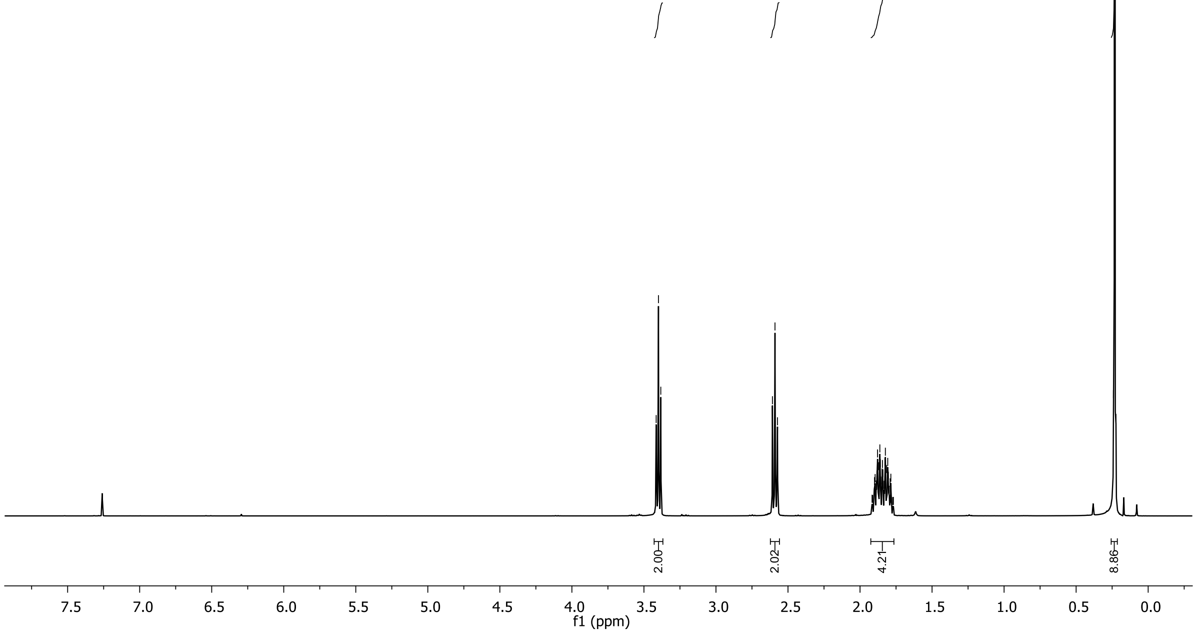
3.41
3.40
3.38
2.61
2.59
2.57
1.88
1.88
1.87
1.86
1.86
1.85
1.84
1.84
1.83
1.82
1.82
1.81
1.81
0.24
0.22
2.00
2.02
4.21
8.86
7.5 7.0 6.5 6.0 5.5 5.0 4.5 4.0 3.5 3.0 2.5 2.0 1.5 1.0 0.5 0.0
f1 (ppm)

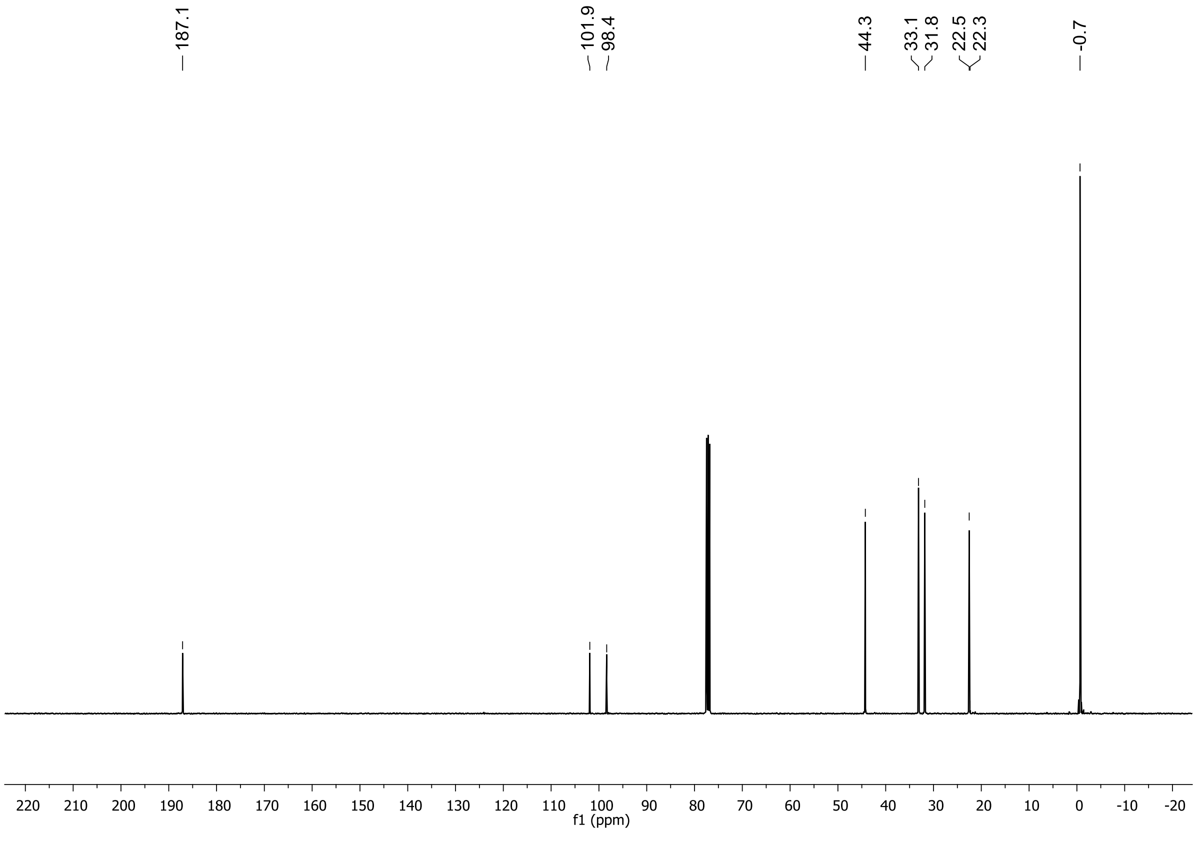
187.1
101.9
98.4
44.3
33.1
31.8
22.5
22.3
-0.7
220 210 200 190 180 170 160 150 140 130 120 110 100 90 80 70 60 50 40 30 20 10 0 -10 -20
f1 (ppm)

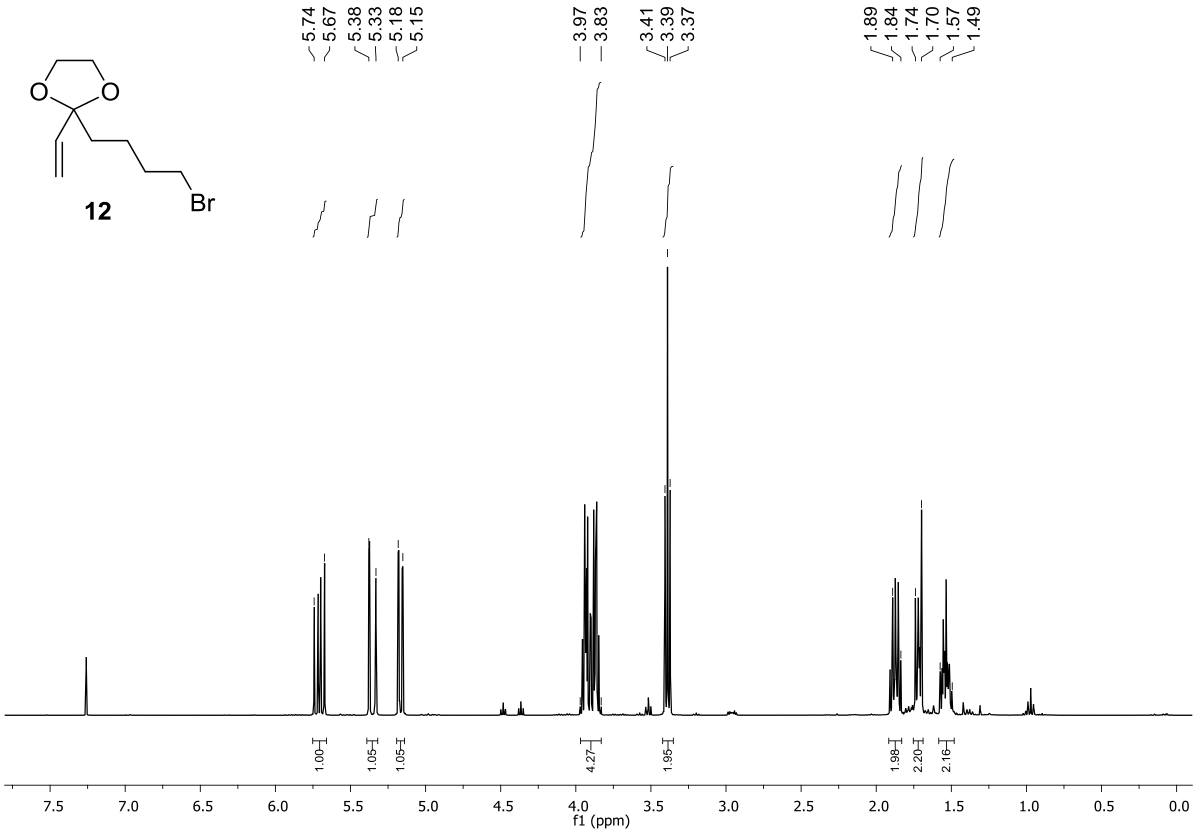
O
O
Br
5.74
5.67
5.38
5.33
5.18
5.15
3.97
3.83
3.41
3.39
3.37
1.89
1.84
1.74
1.70
1.57
1.49
1.00
1.05
1.05
4.27
1.95
1.98
2.20
2.16
7.5 7.0 6.5 6.0 5.5 5.0 4.5 4.0 3.5 3.0 2.5 2.0 1.5 1.0 0.5 0.0
f1 (ppm)

12

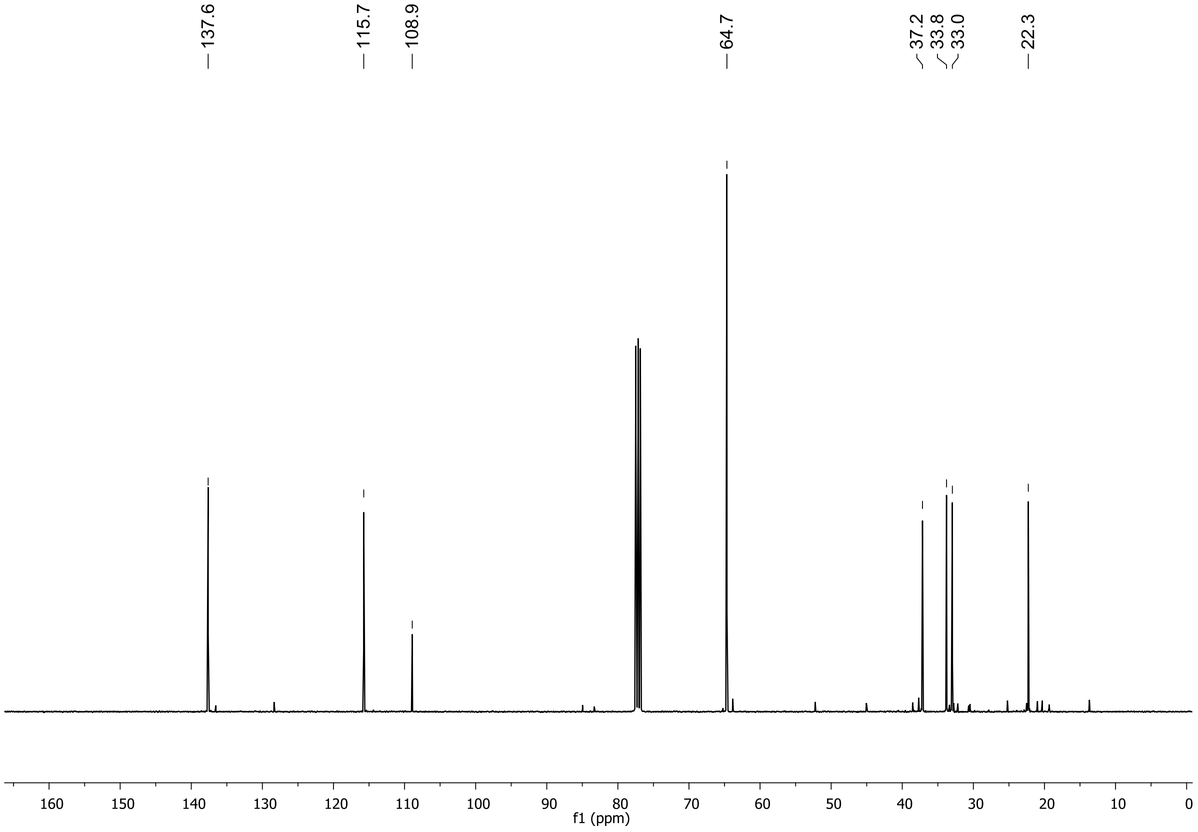
137.6
115.7
108.9
64.7
37.2
33.8
33.0
22.3
160 150 140 130 120 110 100 90 80 70 60 50 40 30 20 10 0
f1 (ppm)

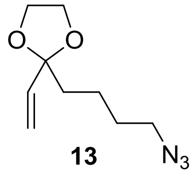
O
O
N3

13

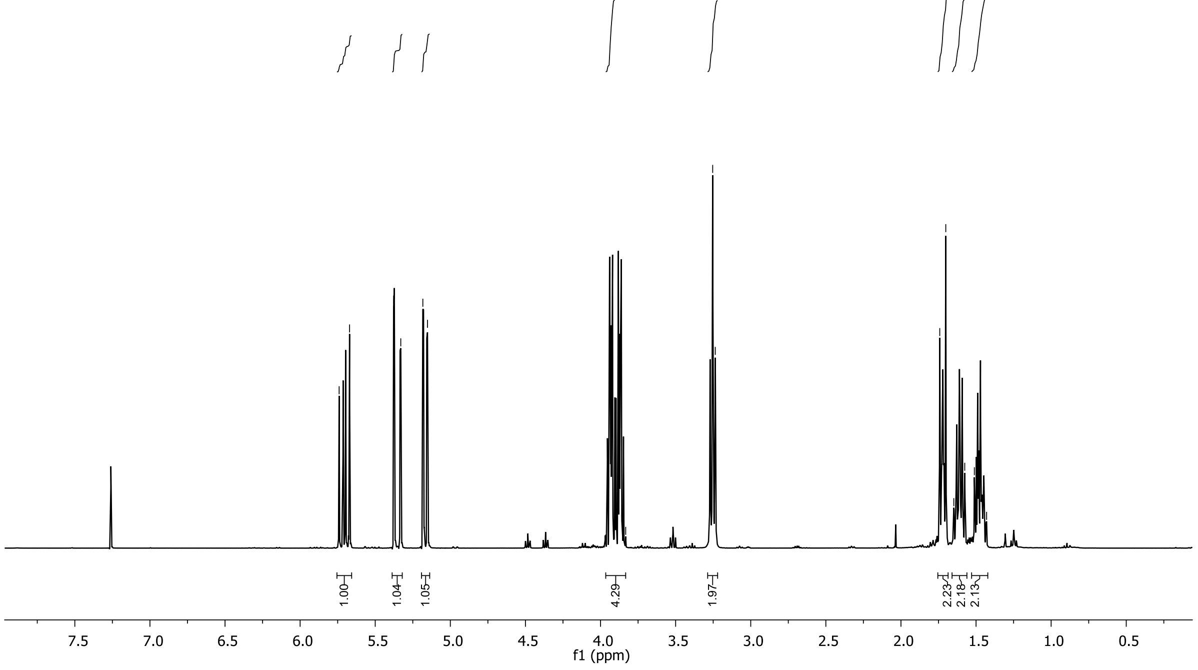
5.74
5.67
5.38
5.33
5.18
5.15
3.97
3.83
3.25
3.24
1.74
1.70
1.65
1.58
1.51
1.43
1.00
1.04
1.05
4.29
1.97
2.23
2.18
2.13
7.5
7.0
6.5
6.0
5.5
5.0
4.5
4.0
3.5
3.0
2.5
2.0
1.5
1.0
0.5
f1 (ppm)

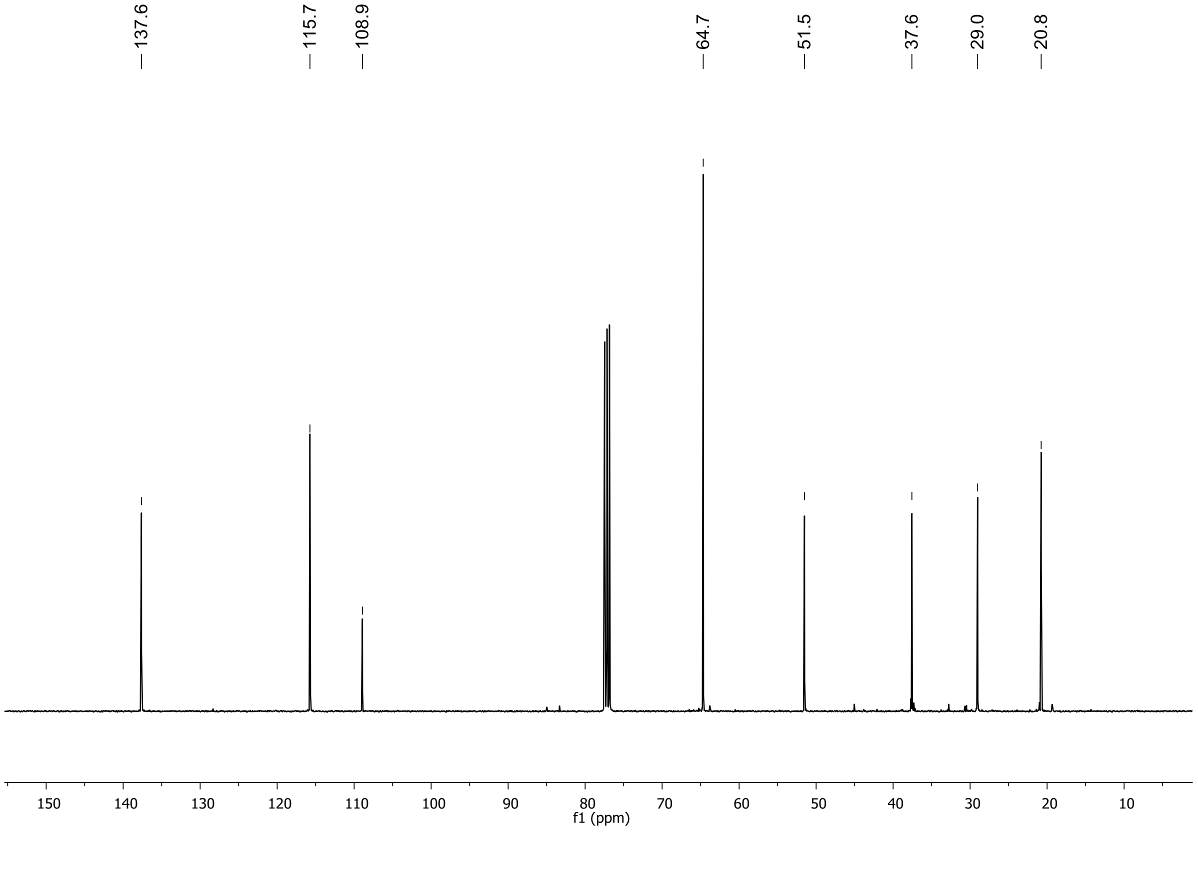
137.6
115.7
108.9
64.7
51.5
37.6
29.0
20.8
150
140
130
120
110
100
90
80
70
60
50
40
30
20
10
f1 (ppm)

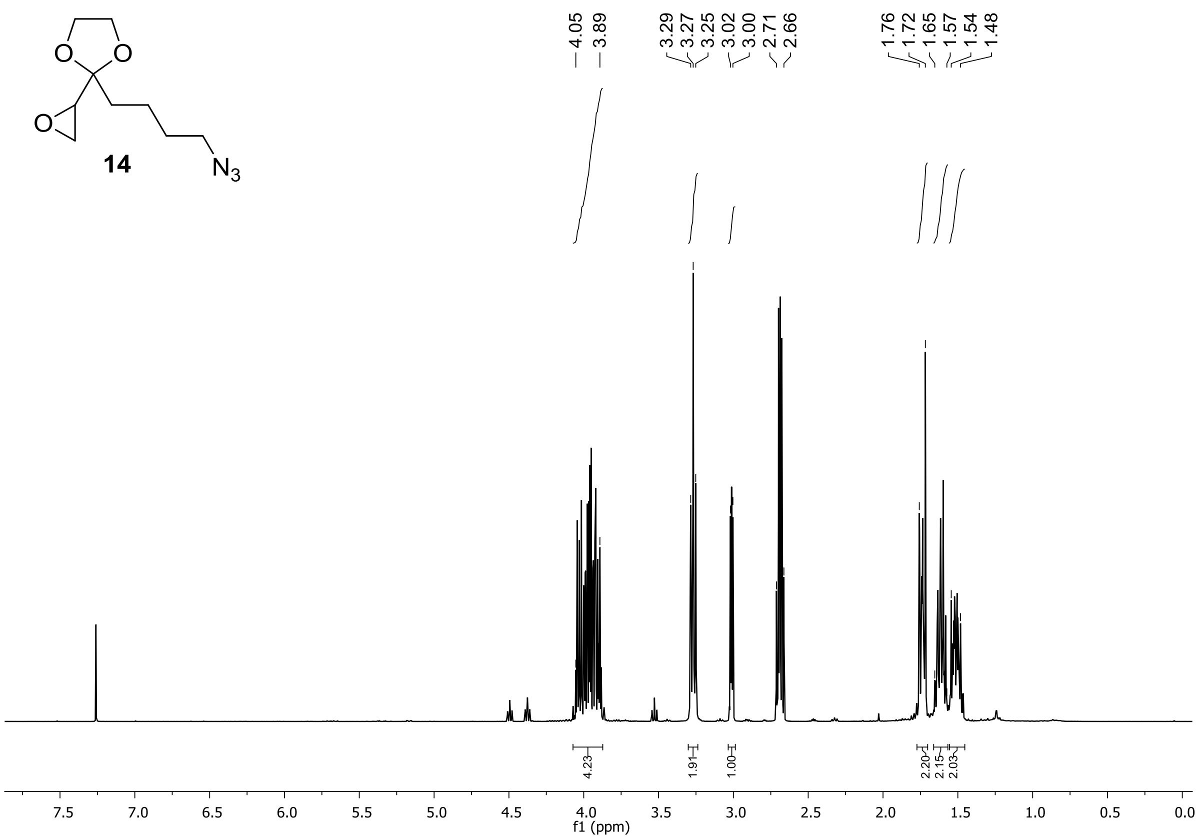
14
N_3
4.05
3.89
3.29
3.27
3.25
3.02
3.00
2.71
2.66
1.76
1.72
1.65
1.57
1.54
1.48
4.23
1.91
1.00
2.20
2.15
2.03
7.5 7.0 6.5 6.0 5.5 5.0 4.5 4.0 3.5 3.0 2.5 2.0 1.5 1.0 0.5 0.0
f1 (ppm)

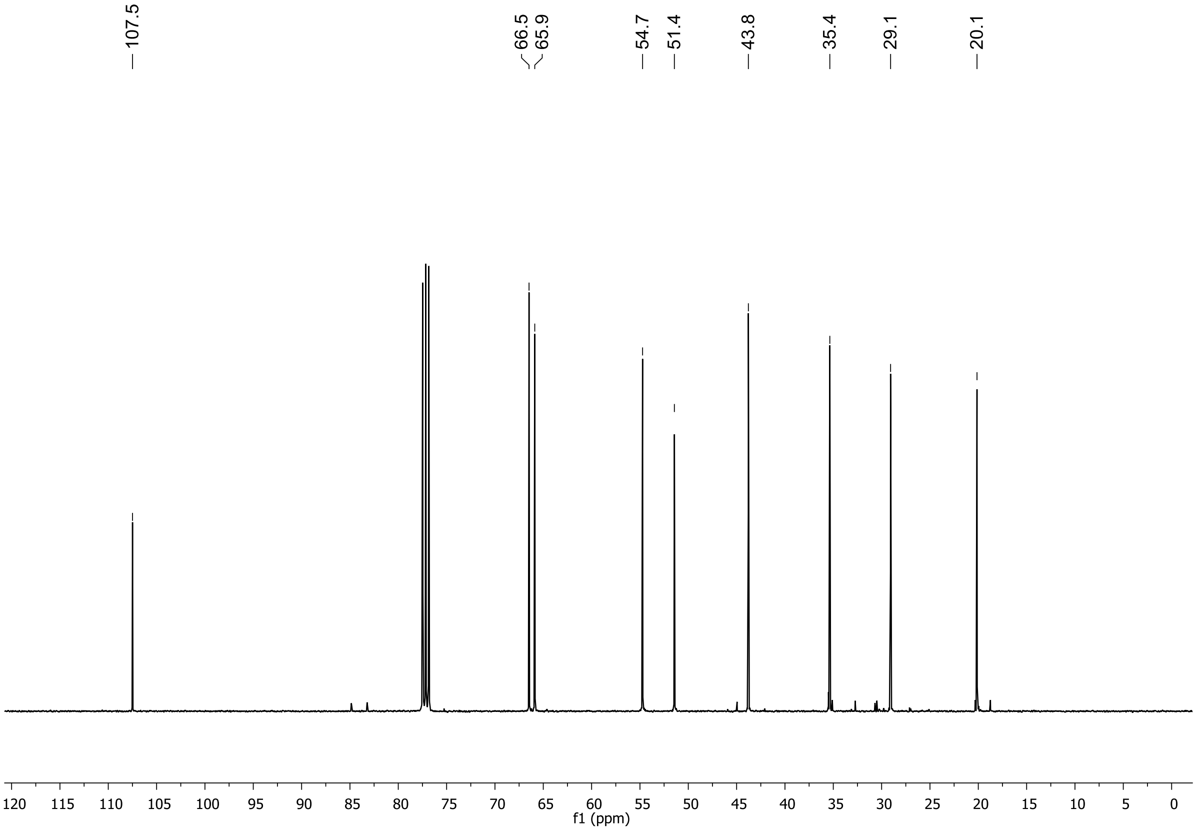
107.5
66.5
65.9
54.7
51.4
43.8
35.4
29.1
20.1
120 115 110 105 100 95 90 85 80 75 70 65 60 55 50 45 40 35 30 25 20 15 10 5 0
f1 (ppm)

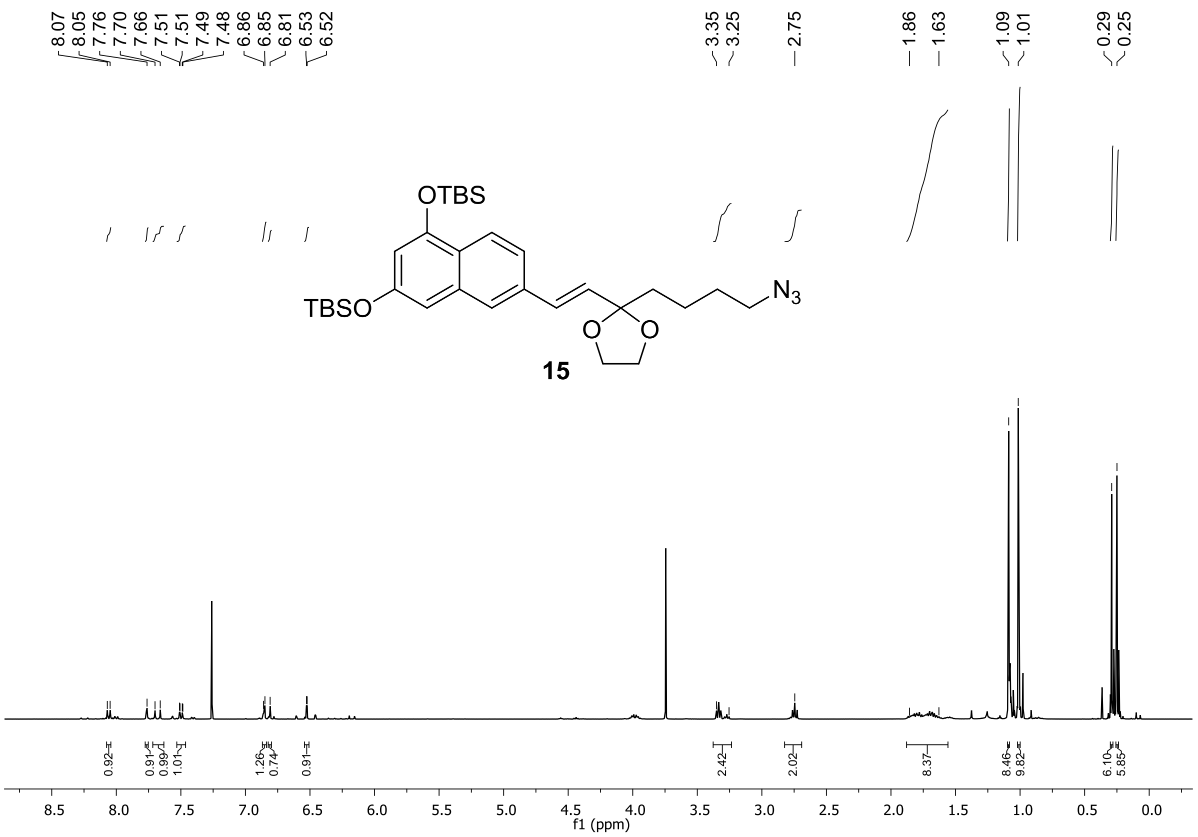
8.07 8.05 7.76 7.70 7.66 7.51 7.51 7.49 7.48 6.86 6.85 6.81 6.53 6.52
3.35 3.25
2.75
1.86 1.63
1.09 1.01
0.29 0.25
OTBS
TBSO
N3
O O
15
0.92 0.91 0.99 1.01 1.26 0.74 0.91 2.42 2.02 8.37 8.46 9.82 6.10 5.85
8.5 8.0 7.5 7.0 6.5 6.0 5.5 5.0 4.5 4.0 3.5 3.0 2.5 2.0 1.5 1.0 0.5 0.0
f1 (ppm)

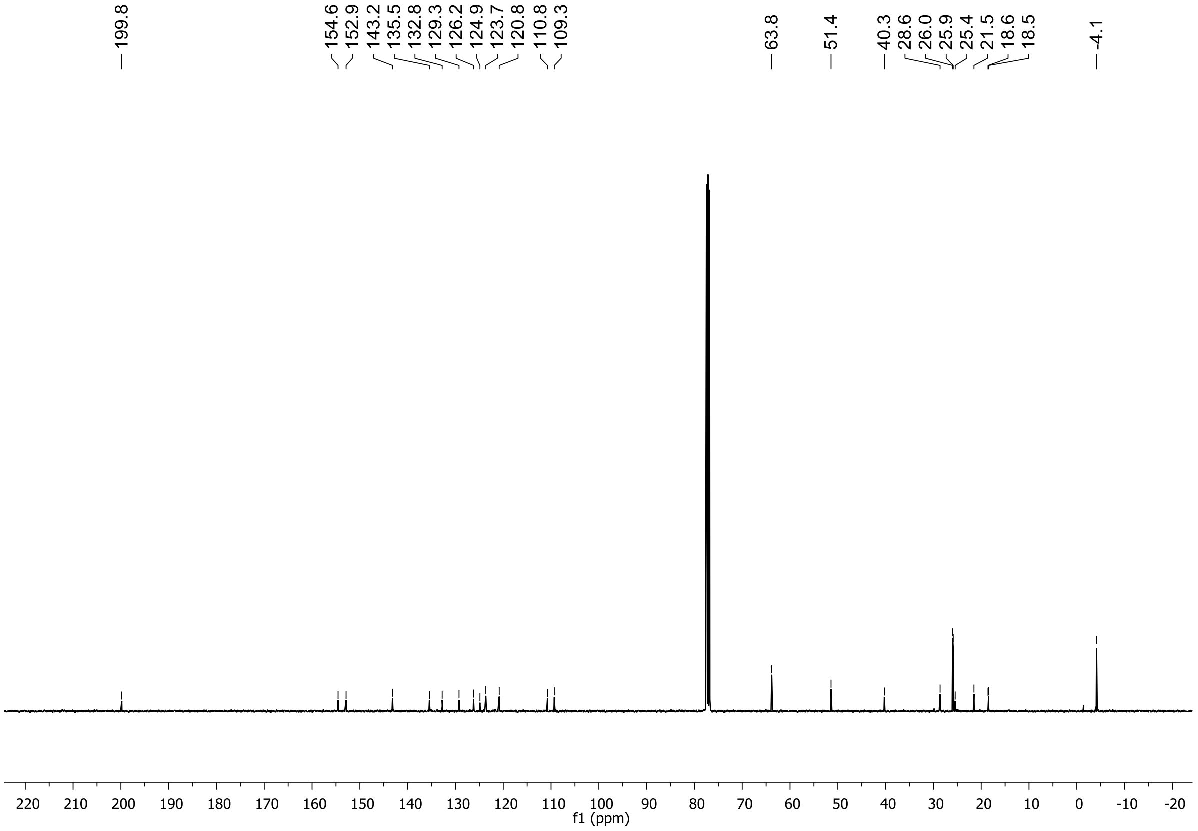
199.8
154.6 152.9 143.2 135.5 132.8 129.3 126.2 124.9 123.7 120.8 110.8 109.3
63.8
51.4
40.3 28.6 26.0 25.9 25.4 21.5 18.6 18.5
-4.1
220 210 200 190 180 170 160 150 140 130 120 110 100 90 80 70 60 50 40 30 20 10 0 -10 -20
f1 (ppm)

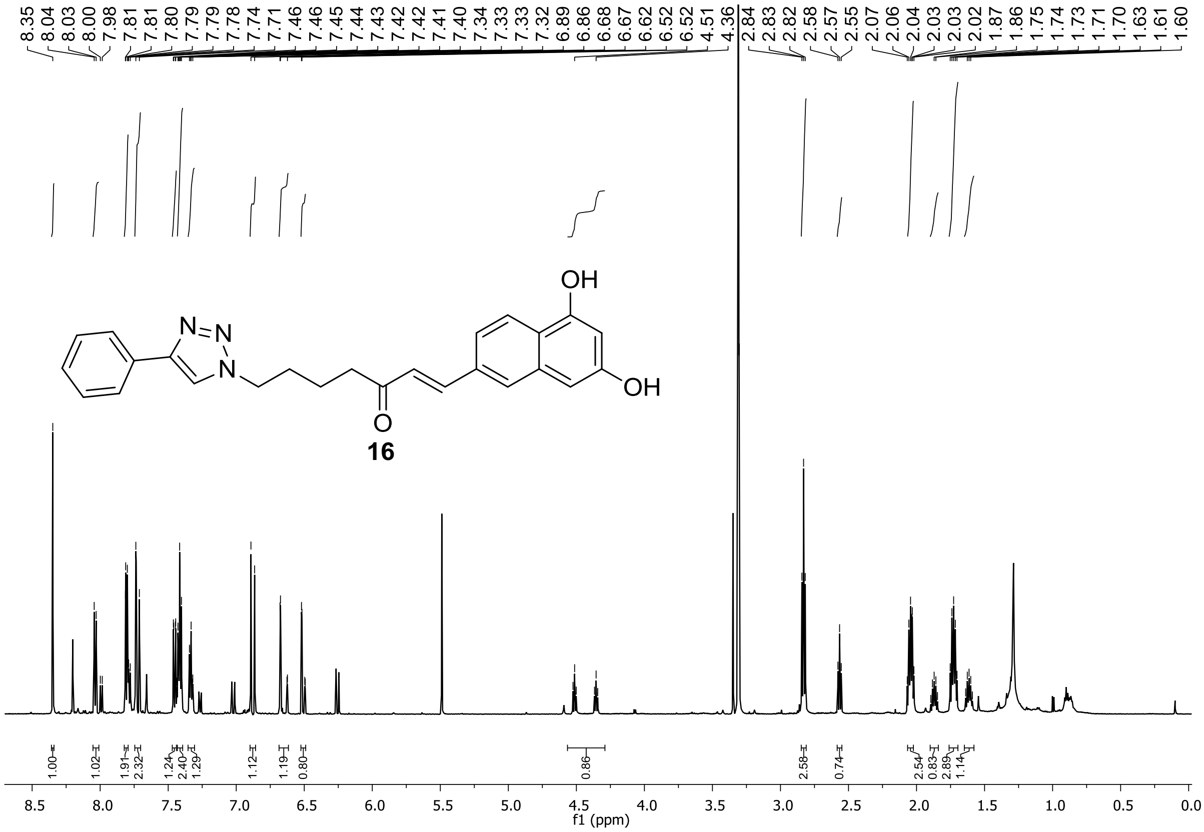
8.35 8.04 8.03 8.00 7.98 7.81 7.81 7.80 7.79 7.79 7.78 7.74 7.71 7.46 7.45 7.44 7.43 7.42 7.42 7.41 7.40 7.34 7.33 7.33 7.32 6.89 6.86 6.68 6.67 6.62 6.52 6.52 4.51 4.36 2.84 2.83 2.82 2.58 2.57 2.55 2.07 2.06 2.04 2.03 2.03 2.02 1.87 1.86 1.75 1.74 1.73 1.71 1.70 1.63 1.61 1.60
OH
N=N
N
OH
O
16
1.00 1.02 1.91 2.32 1.24 2.40 1.29 1.12 1.19 0.80 0.86 2.58 0.74 2.54 0.83 2.89 1.14
f1 (ppm)

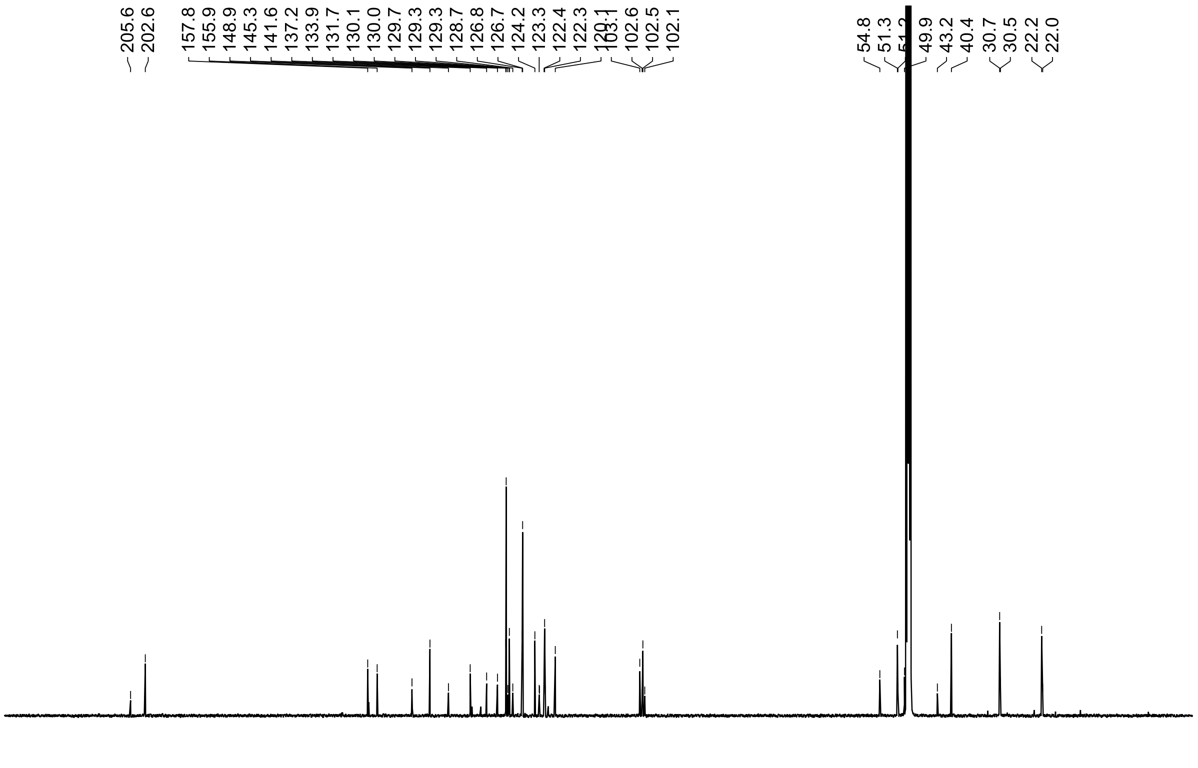
205.6 202.6 157.8 155.9 148.9 145.3 141.6 137.2 133.9 131.7 130.1 130.0 129.7 129.3 129.3 128.7 126.8 126.7 124.2 123.3 122.4 122.3 102.6 102.5 102.1 54.8 51.3 49.9 43.2 40.4 30.7 30.5 22.2 22.0
f1 (ppm)
